# NSUN5 Attenuates Renal Injury and Ferroptosis in Hyperuricaemic Nephropathy Through YBX2‐Dependent Stabilisation of SCD1 m5C Methylation

**DOI:** 10.1002/advs.202521459

**Published:** 2026-04-21

**Authors:** Xiu‐xiu Song, Xiao‐guo Suo, Yue Yu, Kuo Zhang, Chen‐ao Li, Jie Wang, Hui‐xia Xu, Si‐yu Niu, Dong‐xue Lv, Zi‐hao He, Feng‐he Li, Xiao‐ming Meng, Juan Jin

**Affiliations:** ^1^ School of Pharmacy (Anhui Medical University) Inflammation and Immune Mediated Diseases Laboratory of Anhui Province Key Laboratory of Anti‐Inflammatory and Immune Medicine (Anhui Medical University) Ministry of Education Anhui Institute of Innovative Drugs Anhui Medical University Hefei China

**Keywords:** ferroptosis, hyperuricaemic nephropathy, m5C methylation, NSUN5, SCD1

## Abstract

RNA 5‐methylcytosine (m5C) modification plays a critical role in multiple diseases; however, its function in hyperuricaemic nephropathy (HN) remains unclear. Here, we show that renal m5C levels are markedly decreased and the m5C methyltransferase NOP2/Sun RNA methyltransferase 5 (NSUN5) is downregulated in HN mice. Conditional knock‐in (cKI) mice and mouse tubular epithelial cells overexpressing NSUN5 exhibit resistance to uric acid (UA), thereby alleviating kidney injury, inflammation, and fibrosis in HN. Bisulphite sequencing coupled with RNA sequencing identifies stearoyl‐CoA desaturase‐1 (SCD1) as a direct NSUN5 target and reveals the involvement of NSUN5 in ferroptosis regulation. Mechanistically, NSUN5 installs m5C on SCD1 mRNA. The m5C reader YBX2 binds to the modified transcript, prolonging the half‐life of SCD1 mRNA and enhancing its stability, thereby suppressing ferroptosis. Elevated SCD1 also inhibits NF‐κB p65 phosphorylation and nuclear translocation, dampens inflammatory responses and promotes ABCG2‐dependent UA excretion. Recombinant NSUN5 further ameliorates renal injury in HN. Our findings revealed a novel NSUN5‐mediated mechanism and highlight a potential therapeutic target for HN.

## Graphical abstract

NSUN5 is downregulated in hyperuricaemic nephropathy. Overexpression of NSUN5 enhances the stability of SCD1 mRNA through the m5C reader YBX2, ultimately inhibiting ferroptosis in renal tubular epithelial cells. Additionally, this axis suppresses the NF‐κB signaling pathway to alleviate inflammation and upregulates ABCG2 to promote uric acid excretion, thereby effectively mitigating kidney damage and exerting a protective effect.

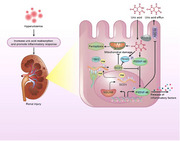

## 1 Introduction

Hyperuricaemia, the fourth most common metabolic disorder after hypertension, hyperlipidaemia, and hyperglycaemia, is characterised by elevated serum uric acid (UA) levels [1]. Long‐term hyperuricaemia promotes the deposition of UA crystals in the kidneys, leading to hyperuricaemic nephropathy (HN) [2], which manifests as tubular injury, inflammatory cell infiltration, and subsequent tubulointerstitial fibrosis [3]. With the acceleration of population ageing and substantial lifestyle changes, the prevalence of hyperuricaemia is increasing annually [4], posing a considerable burden on public health and the economy [5]. Although several potential therapeutic targets have been identified, specific and effective treatments for HN remain lacking. Therefore, elucidating the molecular mechanisms underlying the initiation and progression of HN is essential.

Epigenetic modifications play crucial roles in the development and progression of multiple diseases [6, 7]. Recent studies have revealed that RNA 5‐methylcytosine (m5C) modification plays an important role in the kidney [8]; however, its biological significance and the underlying regulatory mechanisms in HN remain unclear. m5C is a major RNA modification that is widely distributed across various eukaryotic RNAs, including tRNA, and mRNA [9]. Methyltransferases (writer), demethylases (eraser), and recognition proteins (reader) together constitute an enzyme system that dynamically regulates m5C modification [10]. m5C modification in RNA is known to be introduced by the NOL1/NOP2/SUN domain (NSUN) protein family, which includes seven members in humans (NSUN1–7) [11]. It is also demethylated by ten–eleven translocation (TET) [12]. NOP2/Sun RNA methyltransferase 5 (NSUN5) is an RNA methyltransferase that plays diverse roles in physiological and pathological conditions [13]. Studies have reported that downregulation of NSUN5 contributes to inhibiting the p53 pathway, inhibits cell invasion, and suppresses the development of renal cell carcinoma [14]. NSUN5 also mediates m5C modification of FTH1/FTL mRNAs, thereby effectively suppressing ferroptosis in bone marrow mesenchymal stem cells [15]. However, the role of NSUN5 in the development of HN has not yet been elucidated.

In this study, we found that NSUN5 was downregulated in an HN mouse model. Additionally, in vivo and in vitro experiments demonstrated that NSUN5 overexpression significantly suppressed inflammation and fibrosis in HN. Integrated analysis using bisulphite sequencing (BS‐seq) and RNA sequencing (RNA‐seq) confirmed that NSUN5 was involved in the regulation of ferroptosis in HN. Furthermore, NSUN5 overexpression ameliorated the onset and progression of HN by modulating m5C modification of stearoyl‐CoA desaturase‐1 (SCD1) mRNA and enhancing mRNA stability in a Y‐box binding protein 2 (YBX2)‐dependent manner. These findings may provide new strategies for the treatment of HN.

## 2 Results

### 2.1 NSUN5‐Mediated m5C RNA Modification is Reduced in both in vitro and in vivo Models of HN

**FIGURE 1:**
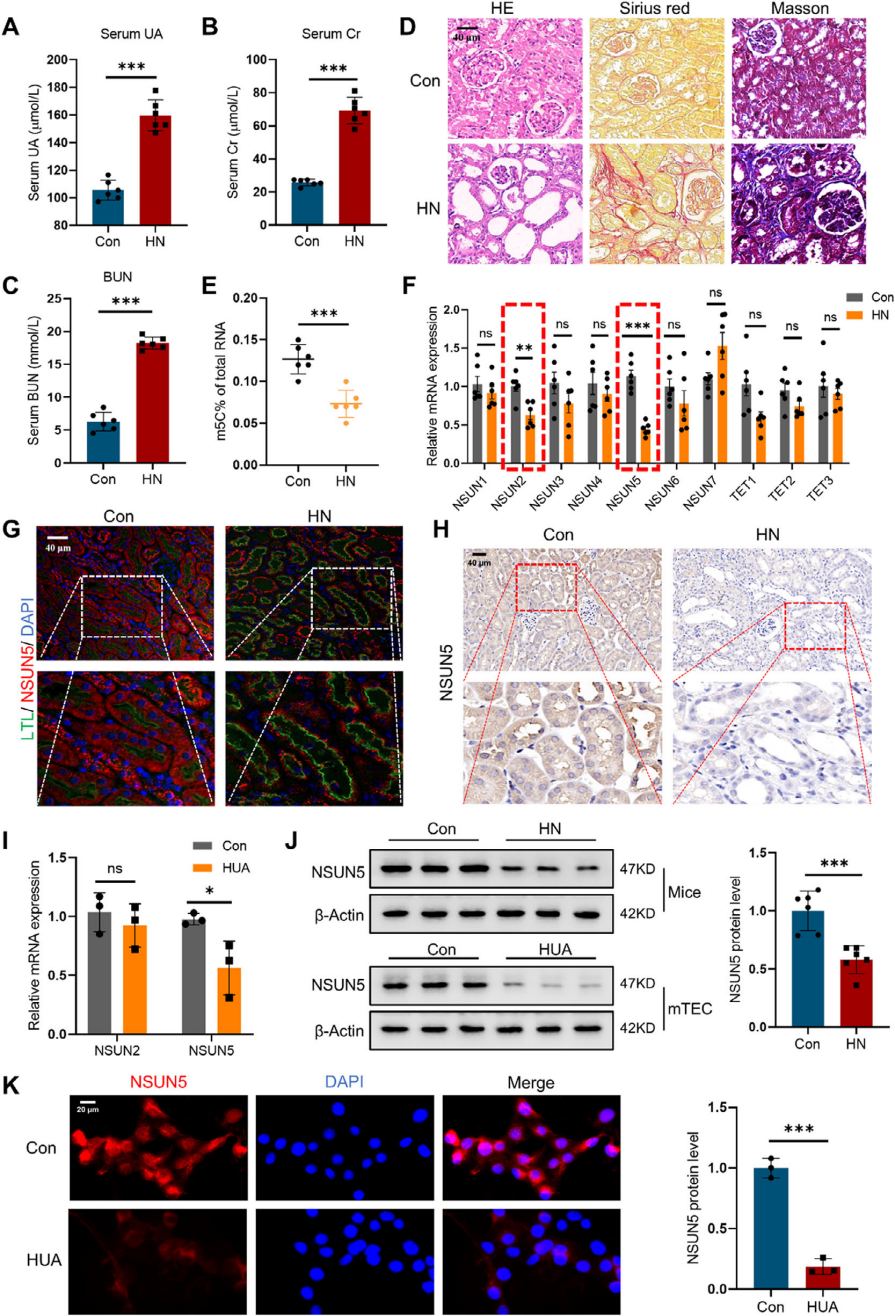
**Reduced 5‐methylcytosine (m5C) levels and decreased NSUN5 expression in HN**. (A–C) Serum uric acid (UA), creatinine (Cr) and blood urea nitrogen (BUN) levels (n = 6). (D) Representative images of haematoxylin and eosin (H&E), Masson's trichrome, and Sirius Red staining. (E) Evaluation of mRNA m5C methylation in an HN mouse model via m5C ELISA (n = 6). (F) Real‐time PCR analysis of m5C regulators (NSUN1–7 and TET1–3) in the HN model (n = 6). (G) Immunofluorescence (IF) analysis of NSUN5 (red) and Lotus tetragonolobus lectin (LTL; green) co‐localisation in kidney tissue. (H) Representative immunohistochemistry (IHC) images of NSUN5 expression in kidneys of HN mice. (I) Real‐time PCR analysis of NSUN2 and NSUN5 mRNA level in mouse tubular epithelial cells (mTECs) (n = 3). (J) Western blot analysis and quantification of NSUN5 protein expression in HN mice and mTECs. (K) Representative IF images of NSUN5 expression in mTECs. Data are presented as the mean ± SEM. *P* values were calculated using two‐tailed unpaired Student's *t*‐tests, ^*^ *p* < 0.05, ^**^ *p* < 0.01, ^***^ *p* < 0.001.

First, we established an HN mouse model. Compared to those in control mice, serum UA levels were significantly elevated in HN mice (Figure 1A). To further evaluate the effect of high UA levels on the kidneys, we measured renal function indicators, including serum creatinine (Cr) and blood urea nitrogen (BUN). The results showed that HN mice exhibited significantly elevated levels of Cr and BUN (Figure 1B, C). In addition, renal pathological staining confirmed that high UA levels induced severe tubular injury and renal fibrosis (Figure 1D). Using an m5C enzyme‐linked immunosorbent assay (ELISA), we observed a significant decrease in RNA m5C levels in the kidney tissue of HN mice (Figure 1E). To determine the mechanism underlying the decrease in m5C RNA modification, we analysed the mRNA level of m5C regulator methyltransferases (NSUN1–7) and demethylases (TET1–3). mRNA level of NSUN2 and NSUN5 was downregulated in HN mice (Figure 1F). However, in a hyperuricaemia cell model (HUA), established by stimulating mouse tubular epithelial cells (mTECs) with UA, only NSUN5 level was persistently downregulated, whereas NSUN2 level remained unchanged (Figure 1I), and NSUN2 overexpression showed no effect on inflammatory response (Figure S1A, B). We then performed immunofluorescence (IF) co‐staining of NSUN5 with the proximal tubule marker LTL (Lotus etragonolobus lectin), the collecting‐duct marker AQP3 (aquaporin 3), and the distal tubule marker calbindin D28 in HN mice. Distinct co‐localisation signals were observed in each case, confirming that NSUN5 was predominantly localized in mTECs (Figure 1G; Figure S1C,D). Immunohistochemistry (IHC), western blotting, and IF further demonstrated that NSUN5 expression was markedly reduced in both in vivo and in vitro models (Figure 1H,J,k). DNA methylation may be involved in the suppression of NSUN5 expression under hyperuricaemic conditions (Figure S1E, F).

### 2.2 NSUN5 Overexpression Ameliorates Inflammation and Fibrosis in HN Mice and UA‐Induced mTECs

**FIGURE 2:**
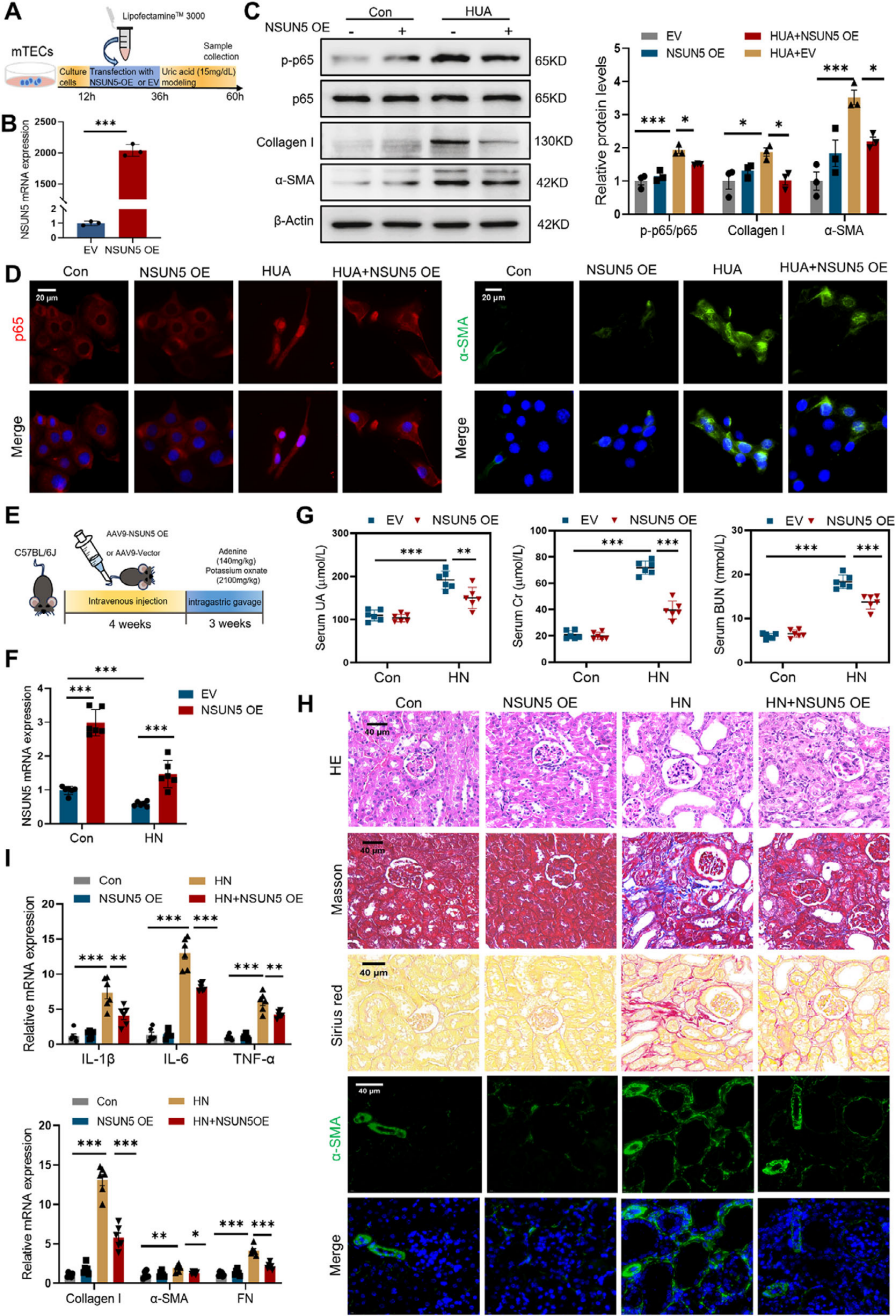
**NSUN5 overexpression attenuates kidney injury in HN mice and uric acid (UA)‐induced mTECs**. (A) Schematic diagram of the in vitro experimental design. (B) Real‐time PCR analysis of NSUN5 mRNA levels (n = 3). (C) Western blot and quantitative analysis of protein expression of p‐p65, collagen I, and α‐SMA (n = 3). (D) IF analysis of p65 and α‐SMA expression in mTECs. (E) Schematic diagram of the in vivo experimental design. (F) Real‐time PCR detection of adeno‐associated virus 9 (AAV9)‐NSUN5 overexpression efficiency. (G) Serum UA, Cr, and BUN levels in each group of mice (n = 6). (H) Representative images of H&E, Masson's trichrome, Sirius Red, and α‐SMA IF staining (n = 6). (I) Real‐time PCR analysis of inflammatory factors (interleukin‐1β [IL‐1β], interleukin‐6 [IL‐6], and tumour necrosis factor alpha [TNF‐α]) and fibrotic markers (collagen I, α‐SMA, and fibronectin [FN]) in HN (n = 6). Data are presented as the mean ± SEM. *P* values were calculated using one‐way ANOVA with Tukey's post hoc test, ^*^ *p* < 0.05, ^**^ *p* < 0.01, ^***^ *p* < 0.001.

To investigate the specific functions of NSUN5 in HN, we constructed an NSUN5 overexpression plasmid (Figure 2A). Following transfection of mTECs with this plasmid, the protein and mRNA levels of NSUN5 were significantly increased (Figure 2B; Figure S2A). Moreover, compared with that in UA‐stimulated mTECs, overexpression of NSUN5 reduced the protein expression and mRNA level of fibrotic markers, including collagen I, alpha‐smooth muscle actin (α‐SMA), and fibronectin (FN) (Figure 2C, D; Figure S2B), and decreased the mRNA levels of pro‐inflammatory factors such as interleukin‐1β (IL‐1β), interleukin‐6 (IL‐6), and tumour necrosis factor alpha (TNF‐α) (Figure S2C). Moreover, elevated NSUN5 expression diminished nuclear p65 abundance and suppressed phosphorylation of NF‐κB p65 (Figure 2C,D; Figure S2D), effectively alleviating UA‐induced inflammatory and fibrotic responses. Consistent with these findings, intravenous injection of the adeno‐associated virus 9 (AAV9)‐NSUN5 virus into mice (Figure 2E) resulted in successful overexpression of NSUN5 in the kidneys (Figure 2F; Figure S3A,B). Morphologically, the kidneys of HN mice appeared pale and uneven; however, upon NSUN5 overexpression, they gradually regained a healthier, ruddy appearance (Figure S3C). Serological indicators showed that NSUN5 overexpression reduced the abnormal increase in serum UA, Cr, and BUN levels caused by HN (Figure 2G). NSUN5 overexpression also significantly alleviated pathological injuries in HN mice, including tubular dilation, and reduced tubular fibrosis, collagen deposition, and the fluorescence intensity of α‐SMA (Figure 2H). In addition, it decreased the mRNA level and protein expression of inflammatory and fibrotic factors (Figure 2I; Figure S3D). Collectively, these results indicate that NSUN5 overexpression effectively improves HN‐induced renal dysfunction and inhibits inflammatory responses and fibrosis.

### 2.3 NSUN5 Overexpression Alleviates Renal Inflammation and Fibrosis Triggered by Spontaneous Hyperuricemia in GLUT9‐Knockout (G9KO) Mice

**FIGURE 3:**
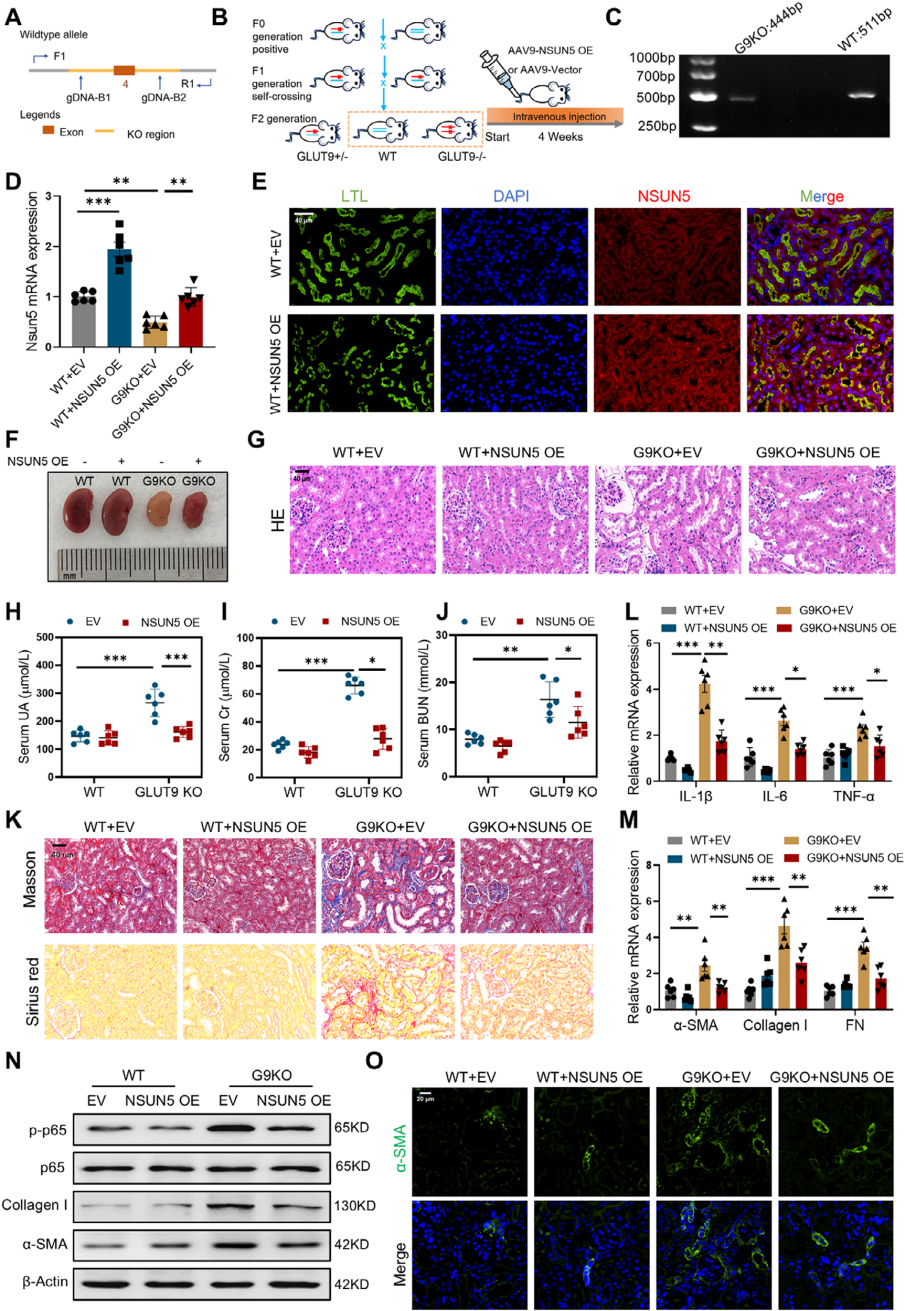
**NSUN5 overexpression attenuates kidney injury in spontaneous hyperuricaemic mice**. (A) Primer design strategy for GLUT9 knockout (G9KO) mice. (B) Breeding of G9KO mice and AAV‐NSUN5 overexpression. (C) Genotyping of mice. (D,E) Real‐time PCR and IF analysis of AAV9‐NSUN5 overexpression efficiency (n = 6). (F) Representative gross morphology of kidneys from G9KO mice. (G) Representative H&E staining of kidney tissues from G9KO mice overexpressing NSUN5 (n = 6). (H–J) Serum UA, Cr, and BUN levels. (K) Representative images of Masson's trichrome and Sirius Red staining (n = 6). (L,M) mRNA levels of inflammatory and fibrotic factors (n = 6). (N) Western blot analysis of p‐p65, collagen I, and α‐SMA expression (n = 6). (O) IF analysis of α‐SMA expression. Data are presented as the mean ± SEM. *P* values were calculated using one‐way ANOVA with Tukey's post hoc test, ^*^ *p* < 0.05, ^**^ *p* < 0.01, ^***^ *p* < 0.001.

To further investigate the role of NSUN5 in UA‐induced kidney disease, we constructed a hyperuricaemia mouse model that spontaneously developed following G9KO (Figure 3A–C). After the G9KO mice reached 8 weeks of age, AAV9‐NSUN5 was injected via the tail vein to increase NSUN5 expression in the kidneys (Figure 3D,E). Consistent with the results from the HN mouse model, NSUN5 overexpression alleviated renal injury in G9KO mice by reducing serum UA, Cr, and BUN levels (Figure 3F–J). Real‐time PCR, western blot, and IF analyses showed that NSUN5 overexpression inhibited the levels of pro‐inflammatory cytokines (IL‐1β, IL‐6, TNF‐α) (Figure 3L), decreased the phosphorylation of NF‐κB p65, and suppressed the mRNA and protein expression of fibrotic markers (α‐SMA, collagen I, FN) (Figure 3M–O). Masson's trichrome and Sirius Red staining revealed that NSUN5 overexpression significantly reduced collagen deposition in G9KO mice (Figure 3K).

### 2.4 Tubule‐Specific NSUN5 Overexpression Alleviates Renal Dysfunction and Fibrosis in HN Mice

**FIGURE 4:**
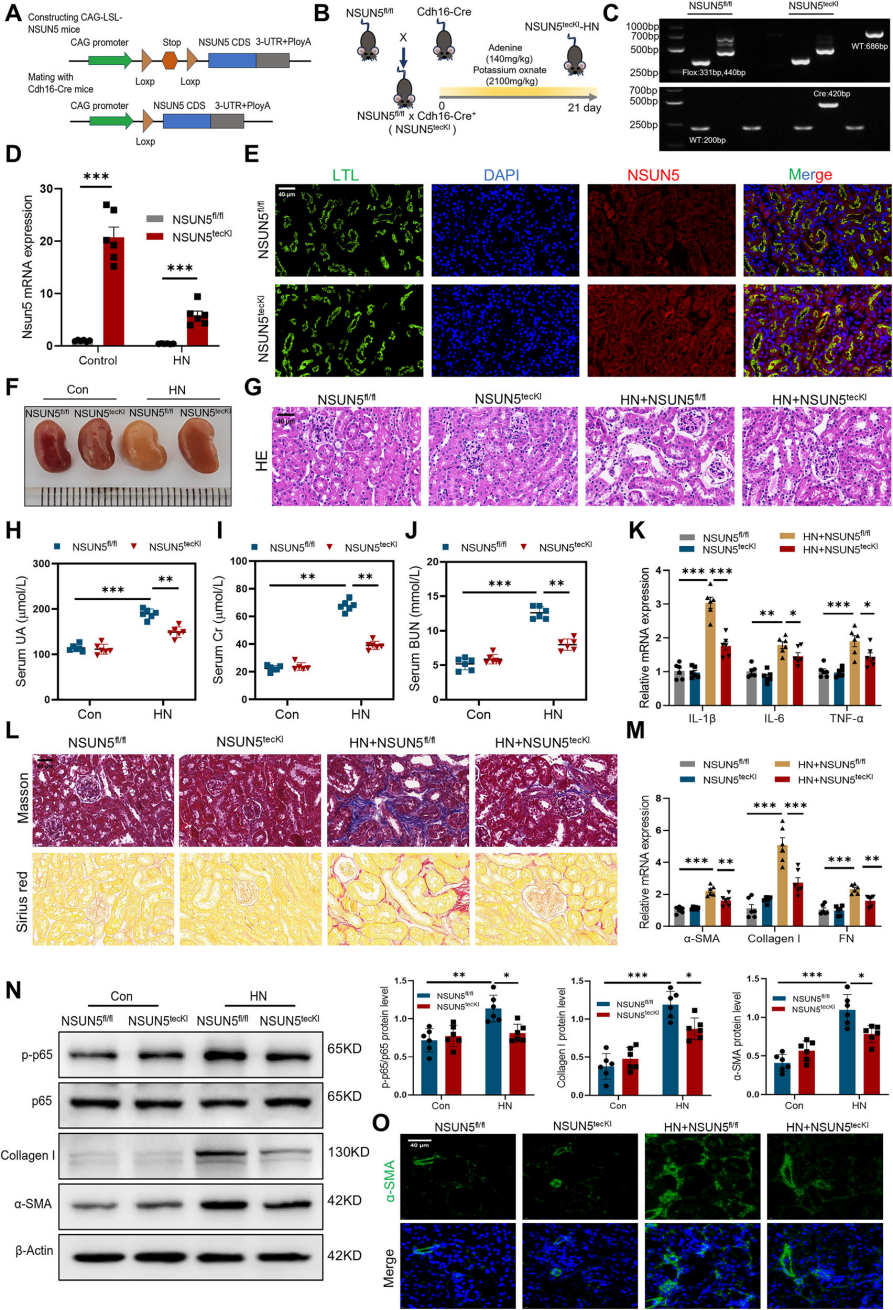
**Tubule‐specific NSUN5 overexpression exerts a protective effect against renal injury in HN mice**. (A) Strategy for generating NSUN5^tecKI^ mice. (B) Experimental design. (C) Genotyping of mice. (D,E) IF and real‐time PCR analysis of NSUN5 overexpression efficiency. (F) Representative gross morphology of the kidneys. (G) Representative H&E staining. (H–J) Serum UA, Cr, and BUN levels (n = 6). (K,M) Real‐time PCR analysis of IL‐1β, IL‐6, TNF‐α, α‐SMA, collagen‐I, and FN (n = 6). (L) Representative images of Masson's trichrome and Sirius Red staining. (N) Western blot analysis of p‐p65, collagen I, and α‐SMA (n = 6). (O) Representative IF staining of α‐SMA. Data are presented as the mean ± SEM. *P* values were calculated using one‐way ANOVA with Tukey's post hoc test, ^*^ *p* < 0.05, ^**^ *p* < 0.01, ^***^ *p* < 0.001.

To investigate the functions of NSUN5 in vivo, we generated renal tubule‐specific NSUN5 overexpression mice (*Cdh16*‐Cre+*NSUN5* ^flox/flox^, designated NSUN5^tecKI^) using transgenic technology (Figure 4A–C). Efficient NSUN5 overexpression in the renal tubules of NSUN5^tecKI^ mice was confirmed (Figure 4D,E; Figure S4A). We performed haematoxylin and eosin (H&E) staining of the heart, liver, spleen, and lungs of NSUN5^fl/fl^ and NSUN5^tecKI^ mice. Histological assessment revealed no pronounced tissue damage in these major organs of NSUN5^tecKI^ mice (Figure S4B). Regarding renal function, no notable differences were observed between NSUN5^tecKI^ and NSUN5^fl/fl^ mice. However, after HN modelling, NSUN5^tecKI^ mice exhibited significant alleviation of renal function impairment (Figure 4H–J), with improved renal tubular lesions and reduced collagen deposition (Figure 4F,G,L). Additionally, the mRNA and protein levels of inflammatory and fibrosis‐related factors were effectively reduced (Figure 4K, M, N). Moreover, the expression of α‐SMA was significantly reduced (Figure 4O; Figure S4C), further confirming that NSUN5 overexpression inhibits renal fibrosis in HN mice. Collectively, these results indicate a key role for NSUN5 in alleviating HN progression and may offer a theoretical basis for developing therapeutic strategies for HN.

### 2.5 SCD1 is a Potential Target of NSUN5

**FIGURE 5:**
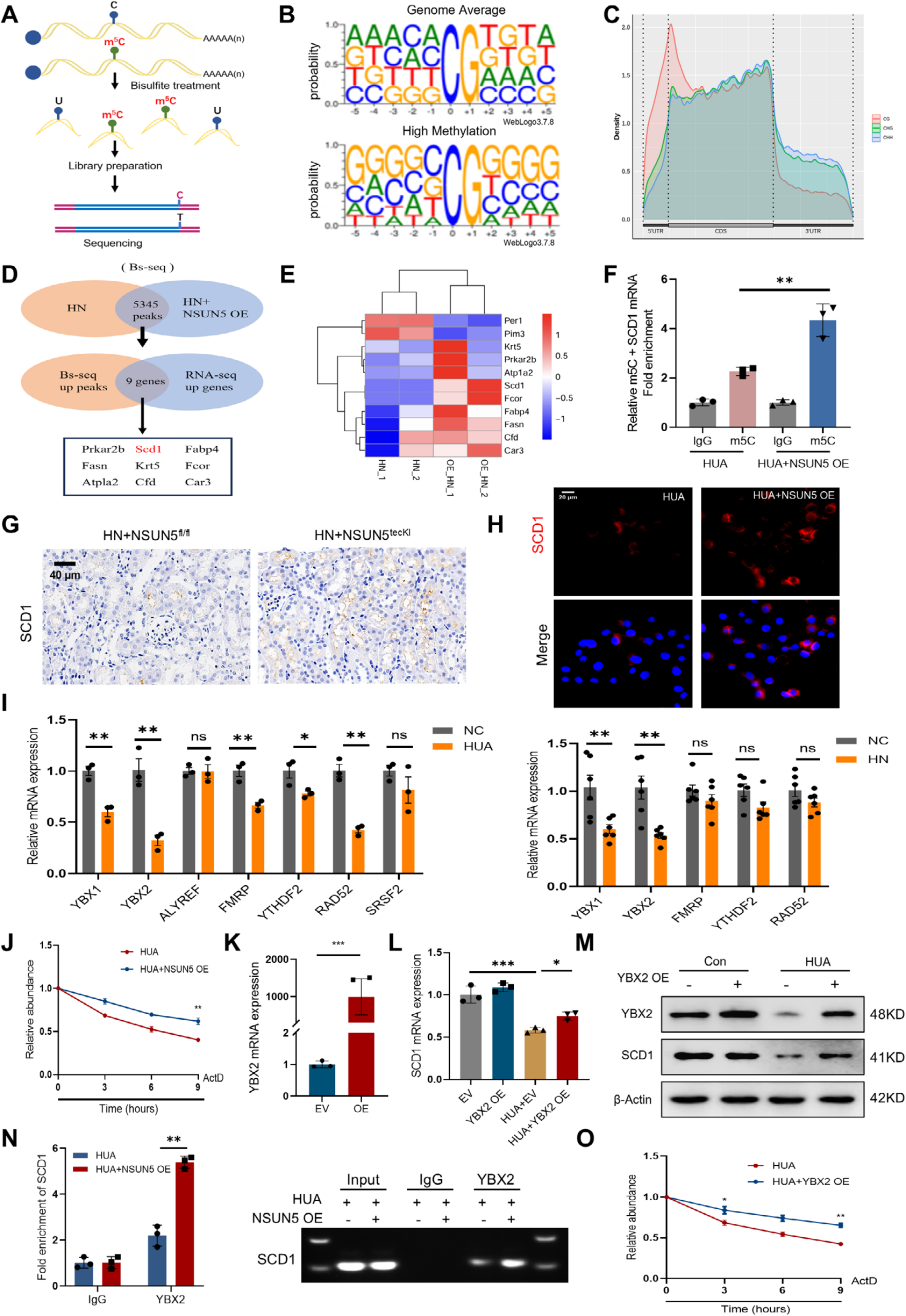
**NSUN5 mediates m5C methylation of SCD1 mRNA in a YBX2‐dependent manner**. (A) Schematic diagram of bisulphite sequencing (BS‐seq). (B) Probability distribution pattern of sequence features within five bases upstream and downstream of m5C sites mediated by NSUN5. (C) The distribution of m5C sites vary across different regions of mRNA. (D,E) Venn diagram and heatmap based on combined analysis of BS‐seq and RNA sequencing (RNA‐seq), showing genes whose m5C modification levels and mRNA levels were simultaneously upregulated by NSUN5 overexpression. (F) Methylated RNA immunoprecipitation (MeRIP)‐qPCR analysis of changes in m5C modification of the SCD1 mRNA in mTECs stimulated by UA (n = 3). (G,H) IHC and IF analyses in vivo and in vitro confirmed that NSUN5 overexpression reversed the reduction in SCD1 expression in HN. (I) Real‐time PCR analysis of YBX1, YBX2, ALYRER, FMRP, YTHDF2, RAD52, and SRSF2 level. (J) Decay rate of *SCD1* mRNA in NSUN5‐overexpressing mTECs following treatment with actinomycin D. (K) Real‐time PCR analysis of YBX2 overexpression efficiency following plasmid transfection (n = 3). (L,M) Real‐time PCR and western blot analyses of SCD1 expression (n = 3). (N)RIP‐qPCR assays of the binding of SCD1 mRNA to the YBX2 in the presence or absence of NSUN5 OE treatment (n = 3). (O) Decay rate of SCD1 mRNA in YBX2‐overexpressing mTECs following treatment with actinomycin D. Data are presented as the mean ± SEM. An unpaired two‐tailed Student's *t*‐test was used to compare the means of two groups. Multiple comparisons were performed using one‐way ANOVA with Tukey's post hoc test. ^*^ *p* < 0.05, ^**^ *p* < 0.01, ^***^ *p* < 0.001.

To further investigate the downstream mechanisms of NSUN5 regulation in HN, we performed BS‐seq (Figure 5A) and RNA‐seq on the kidneys of HN mice with and without NSUN5 overexpression. A probability pattern diagram generated by WebLogo showed that the sequence C[A/C]GGGG downstream of the m5C sites had a significantly higher frequency (Figure 5B). The BS‐seq results also indicated that m5C peaks were highly enriched in the 5'UTR and CDS regions (Figure 5C). To identify downstream target genes regulated by NSUN5, we conducted a cross‐analysis of “genes with significantly upregulated m5C methylation levels” in BS‐seq and “genes with significantly upregulated mRNA levels” in RNA‐seq. We found that m5C modification of SCD1 mRNA was highly correlated with NSUN5 expression (Figure 5D,E). Notably, compared with that in normal mice, SCD1 mRNA level was markedly downregulated in HN mice, whereas another gene, FCOR, showed no significant change (Figure S5A), indicating that SCD1 is a potential target for further study. Methylated RNA immunoprecipitation (MeRIP)‐qPCR analysis showed that NSUN5 overexpression significantly enhanced the enrichment of m5C antibodies on SCD1 mRNA (Figure 5F). To verify whether the methyltransferase activity of NSUN5 is required for its regulatory function, we generated an enzymatically dead double mutant by simultaneously replacing two conserved catalytic cysteine residues, C308 and C359, with alanine (C308A/C359A) [16]. In UA‐induced mTECs overexpressing either wild‐type (NSUN5‐WT) or mutant (NSUN5‐mut) NSUN5, only NSUN5‐WT increased the m5C modification level on SCD1 mRNA, whereas NSUN5‐mut had no effect (Figure S5B). In addition, IHC, real‐time PCR, and IF analyses revealed that NSUN5 overexpression significantly reversed the downregulation of SCD1 in HN or UA‐induced mTECs (Figure 5G,H; Figure S5C‐E). Collectively, these results suggest that SCD1 is a key downstream target of NSUN5 in HN.

### 2.6 YBX2 Enhances the Stability of SCD1 mRNA in an m5C‐Dependent Manner

In addition to methyltransferases and demethylases, m5C readers play an important role in m5C RNA modification. Real‐time PCR showed that the mRNA levels of YBX1 and YBX2 decreased in HN mice and UA‐induced mTECs. Notably, YBX2 showed a more significant reduction in HUA group cells (Figure 5I). Previous studies have shown that YBX2 regulates mRNA stability [17, 18]. Stability assays revealed that NSUN5 overexpression prolonged the half‐life of SCD1 mRNA under UA conditions, suggesting that m5C modification of SCD1 promotes mRNA stability (Figure 5J). To confirm whether YBX2 could stabilize SCD1 mRNA, we constructed a YBX2‐specific overexpression plasmid and confirmed its efficiency (Figure 5K). YBX2 overexpression markedly increased both SCD1 mRNA and protein levels (Figure 5L,M; Figure S5F). Next, RIP with a YBX2 antibody demonstrated that endogenous YBX2 bound to SCD1 mRNA (Figure 5N). In addition, we used the transcription inhibitor actinomycin D (5 µg/mL) and found that YBX2 overexpression significantly extended the half‐life of SCD1 mRNA (Figure 5O). Collectively, these results indicate that YBX2 recognizes and stabilizes m5C‐modified SCD1 mRNA.

### 2.7 SCD1 Inhibits UA‐Induced Pro‐Inflammatory and Pro‐Fibrotic Responses in mTECs

**FIGURE 6:**
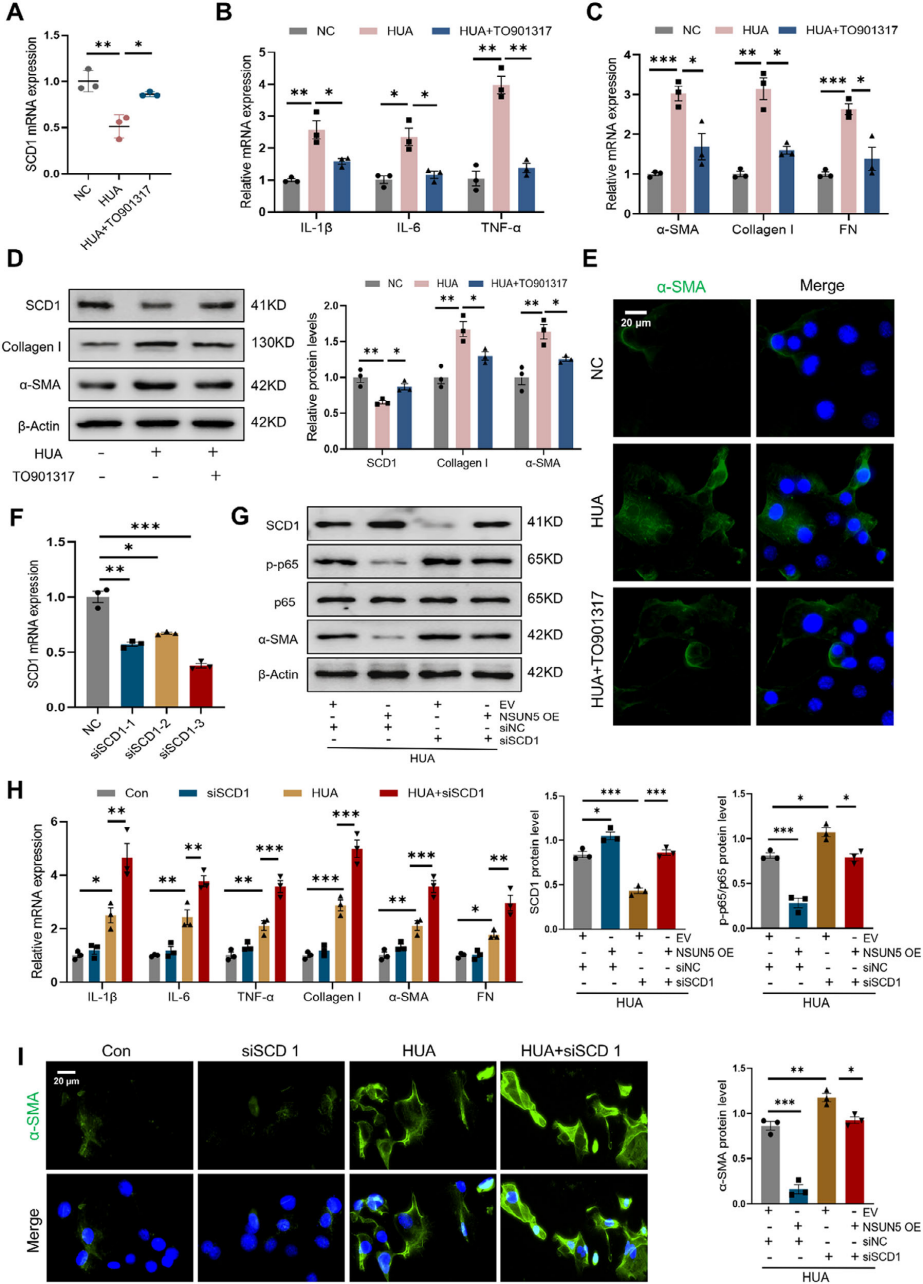
**SCD1 inhibits inflammation and fibrosis in UA‐induced mTECs**. (A) Levels of SCD1 mRNA (n = 3). (B,C) Real‐time PCR analysis of IL‐1β, IL‐6, TNF‐α, α‐SMA, collagen I, and FN in vitro (n = 3). (D) Protein expression of SCD1, collagen I, and α‐SMA (n = 3). (E) IF analysis of α‐SMA expression. (F) Real‐time PCR analysis evaluating the efficacy of SCD1 silencing (n = 3). (G) Western blot analysis of p‐p65, SCD1, and α‐SMA in mTECs (n = 3). (H) Real‐time PCR analysis of inflammatory and fibrotic factors. (I) IF analysis of α‐SMA expression upon SCD1 silencing. Data are presented as the mean ± SEM. *P* values were calculated using one‐way ANOVA with Tukey's post hoc test, ^*^ *p* < 0.05, ^**^ *p* < 0.01, ^***^ *p* < 0.001.

To further investigate the role of SCD1 in HN, we first treated mTECs with the liver X receptor agonist TO901317 (10 µm), which upregulates SCD1 expression [19]. Treatment of UA‐induced mTECs with TO901317 significantly increased SCD1 mRNA level (Figure 6A) and significantly reduced that of inflammatory factors (IL‐1β, IL‐6, and TNF‐α) (Figure 6B) as well as the mRNA and protein expression of α‐SMA, collagen I, and FN (Figure 6C,D). IF analysis confirmed these results, showing a significant decrease in α‐SMA expression following TO901317 treatment (Figure 6E). These findings indicate elevated SCD1 effectively inhibits inflammatory responses and fibrotic processes in UA‐induced mTECs.

Additionally, we designed and screened small interfering RNAs (siRNAs) targeting SCD1 and selected the most effective sequence for subsequent transfections (Figure 6F). SCD1 knockdown in UA‐induced mTECs increased the mRNA level and protein expression of pro‐inflammatory and fibrotic factors (Figure 6H; Figure S6A), and IF results were consistent with these findings (Figure 6I). Additionally, silencing SCD1 in NSUN5‐overexpressing mTECs markedly enhanced NF‐κB p65 phosphorylation and promoted the production of multiple fibrotic and inflammatory factors (Figure 6G; Figure S6B), thereby reversing the protective effect of NSUN5 against HN. Collectively, these findings confirm that decreased SCD1 expression promotes UA‐induced inflammation and fibrosis.

### 2.8 SCD1 Reduces UA Levels by Upregulating ABCG2 Expression via Inhibition of NF‐κB Nuclear Translocation in UA‐Induced mTECs

**FIGURE 7:**
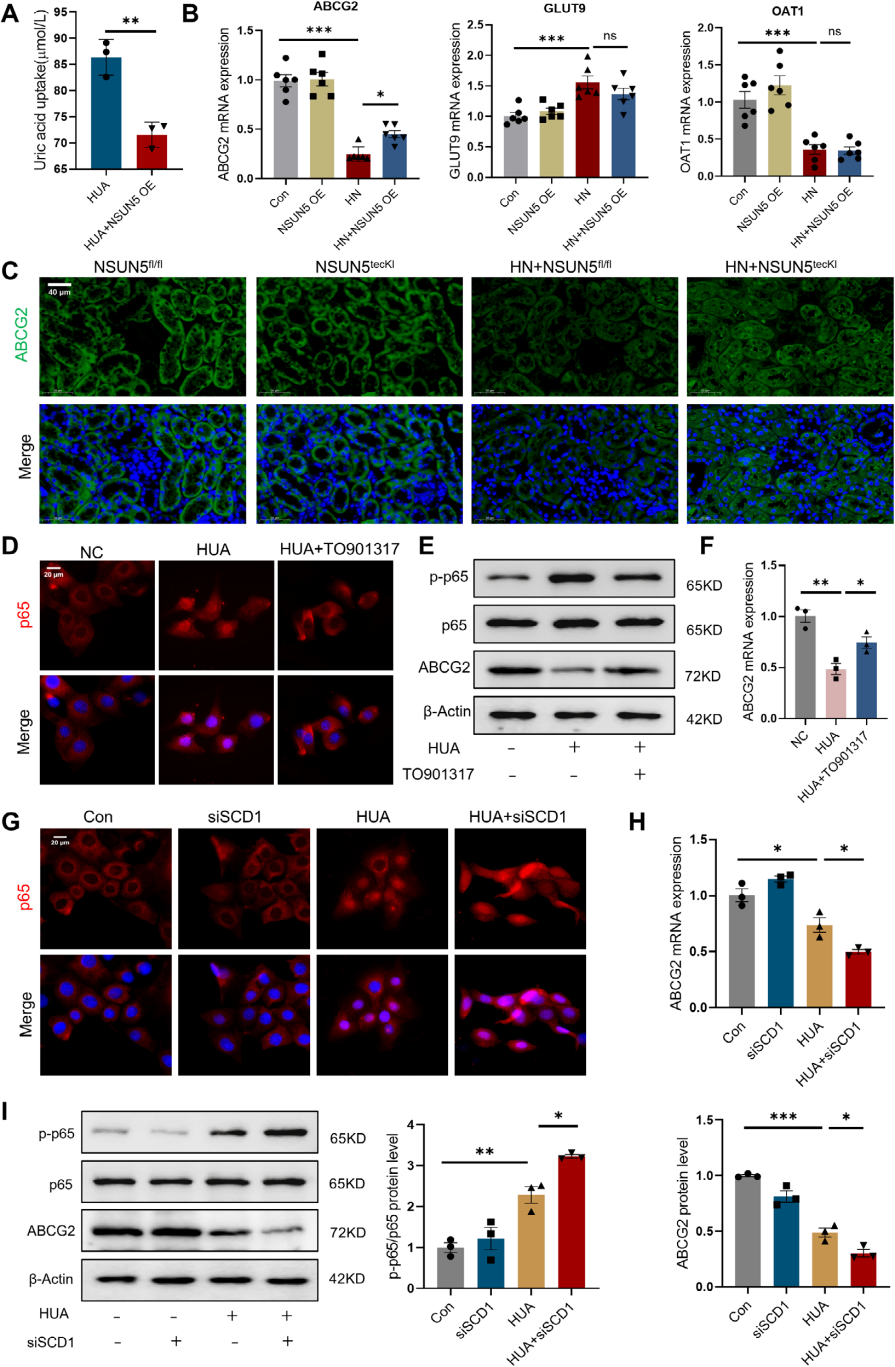
**SCD1 restores ABCG2 expression by inhibiting NF‐κB nuclear translocation**. (A) UA uptake (n = 3). (B) Real‐time PCR analysis of ABCG2, GLUT9, and OAT1 in HN mice (n = 6). (C) IF analysis of ABCG2 expression. (D–G) IF analysis of p65 expression in mTECs. (E,F) Western blot and real‐time PCR analyses of p‐p65 and ABCG2 in mTECs treated with TO901317 (n = 3). (H,I) Western blot and real‐time PCR analysis of p‐p65 and ABCG2 in mTECs with SCD1 silenced (n = 3). Data represented as the mean ± SEM. *P*‐values are calculated using one‐way ANOVA with Tukey's post hoc test, ^*^ *p* < 0.05, ^**^ *p* < 0.01, ^***^ *p* < 0.001.

In UA uptake assays, NSUN5 overexpression markedly reduced intracellular UA levels (Figure 7A). As urate transporters are pivotal for UA homeostasis by regulating reabsorption and secretion, we quantified the mRNA levels of several such transporters. NSUN5 overexpression robustly upregulated ATP binding cassette subfamily G member 2 (ABCG2), both in vivo and in vitro (Figure 7B; Figure S7A), a finding confirmed via IF (Figure 7C). Moreover, NSUN5 overexpression reduced NF‐κB p65 phosphorylation (Figure 4N) and blocked its nuclear translocation (Figure 2D; Figure S2D). However, the underlying mechanisms remain unclear. One previous study indicates that SCD1 deficiency activates the NF‐κB pathway [20], leading us to hypothesize that SCD1 mediates the regulation of NF‐κB by NSUN5. Western blot analysis showed that TO901317 treatment markedly suppressed p65 phosphorylation in UA‐stimulated mTECs (Figure 7E; Figure S7B), whereas SCD1 knockdown increased it (Figure 7I). IF analysis further revealed that TO901317 inhibited UA‐induced p65 nuclear translocation (Figure 7D), whereas SCD1 knockdown promoted it (Figure 7G), underscoring the pivotal role of SCD1 in controlling NF‐κB p65 phosphorylation and nuclear import. Our previous work demonstrates that NF‐κB inhibition robustly upregulates the UA transporter ABCG2 [21]. Consistently, TO901317 enhanced ABCG2 mRNA level and protein expression in UA‐stimulated mTECs (Figure 7E,F; Figure S7B), whereas SCD1 silencing suppressed them (Figure 7H,I), confirming NF‐κB‐mediated control of ABCG2. Collectively, NSUN5 upregulates SCD1, thereby suppressing NF‐κB p65 phosphorylation and nuclear translocation, alleviating inflammation, and increasing ABCG2 expression to promote UA excretion—ultimately exerting protective effects against HN.

### 2.9 NSUN5‐Mediated m5C Modification of SCD1 mRNA Suppresses Ferroptosis in HN

**FIGURE 8:**
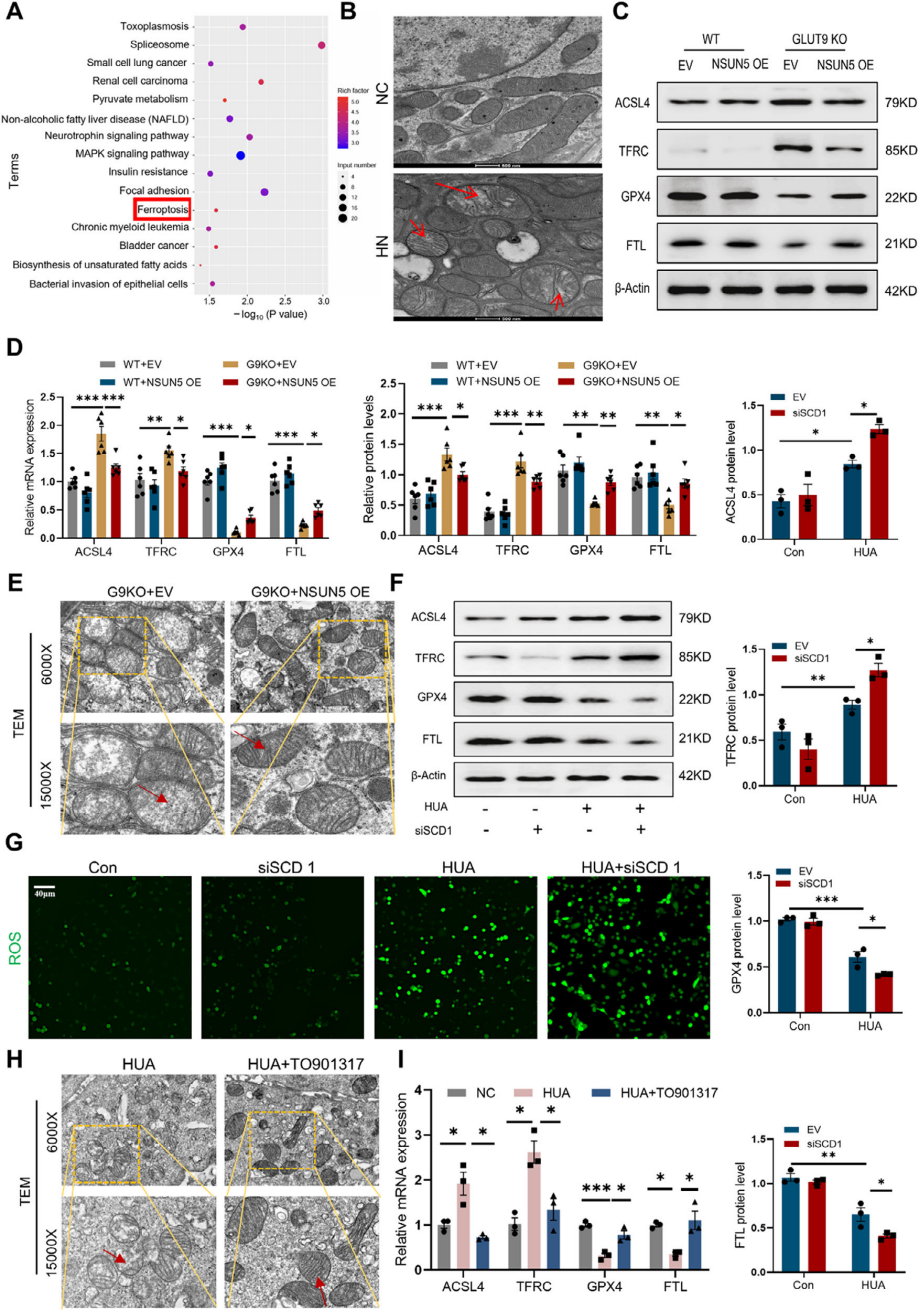
**NSUN5 inhibits ferroptosis through m5C methylation of *SCD1* mRNA in HN mice and UA‐induced mTECs**. (A) KEGG pathway enrichment analysis diagram. (B) Representative transmission electron microscopy (TEM) images of kidneys from different groups. (C) Western blot analysis of ACSL4, TFRC, FTL, and GPX4 expression in G9KO mice (n = 6). (D) Real‐time PCR analysis of ACSL4, TFRC, FTL, and GPX4 level in G9KO mice (n = 6). (E) Representative TEM images of kidneys from G9KO mice. (F) Western blot analysis of ACSL4, TFRC, FTL, and GPX4 in mTECs following SCD1 silencing (n = 3). (G) Reactive oxygen species (ROS) staining. (H) Representative TEM images of mTECs. (I) Real‐time PCR analysis of ACSL4, TFRC, FTL, and GPX4 in mTECs treated with TO901317 (n = 3). Data are presented as the mean ± SEM. *P* values were calculated using one‐way ANOVA with Tukey's post hoc test, ^*^ *p* < 0.05, ^**^ *p* < 0.01, ^***^ *p* < 0.001.

Using KOBAS 3.0, we performed a pathway enrichment analysis of the differentially methylated genes and found that ferroptosis was significantly enriched (Figure 8A). To further explore the effect of NSUN5 on ferroptosis in HN mice, we observed the morphological features of ferroptosis using transmission electron microscopy (TEM). Mitochondrial cristae were reduced or lost, and mitochondrial membrane density was increased in the kidney tissue of HN mice (Figure 8B), whereas overexpression of NSUN5 effectively alleviated these mitochondrial morphological abnormalities (Figure 8E). Furthermore, the protein and mRNA levels of pro‐ferroptotic markers ACSL4 and TFRC were significantly upregulated, whereas the expression of anti‐ferroptotic markers GPX4 and FTL was downregulated in G9KO mice. In contrast, overexpression of NSUN5 significantly reversed these effects (Figure 8C,D). To investigate whether NSUN5 exerts its anti‐ferroptosis mechanism via m5C modification of SCD1, we transfected siRNA into mTECs to silence SCD1. Real‐time PCR and western blot analyses showed that SCD1 knockdown significantly exacerbated UA‐induced ferroptosis (Figure 8F; Figure S8A). Additionally, reactive oxygen species (ROS) staining showed a significant increase in oxidized lipids (intensified green fluorescence) after 24 h of UA stimulation, which was further amplified by SCD1 depletion (Figure 8G). These results indicate that loss of SCD1 promotes UA‐driven ferroptosis and suggest that NSUN5 alleviates ferroptosis in HN by upregulating SCD1. To confirm this, we treated mTECs with TO901317, which markedly ameliorated the UA‐induced ferroptotic phenotypes (Figure 8H), downregulated ACSL4 and TFRC, upregulated GPX4 and FTL (Figure 8I; Figure S8C), and reduced ROS levels (Figure S8B), confirming that activation of SCD1 effectively suppresses ferroptosis. Taken together, our findings indicate that NSUN5 protects the kidney against hyperuricaemia‐induced ferroptosis by modulating m5C methylation of SCD1 mRNA.

### 2.10 Exogenous NSUN5 Administration Protects Against Renal Injury in HN Mice

**FIGURE 9:**
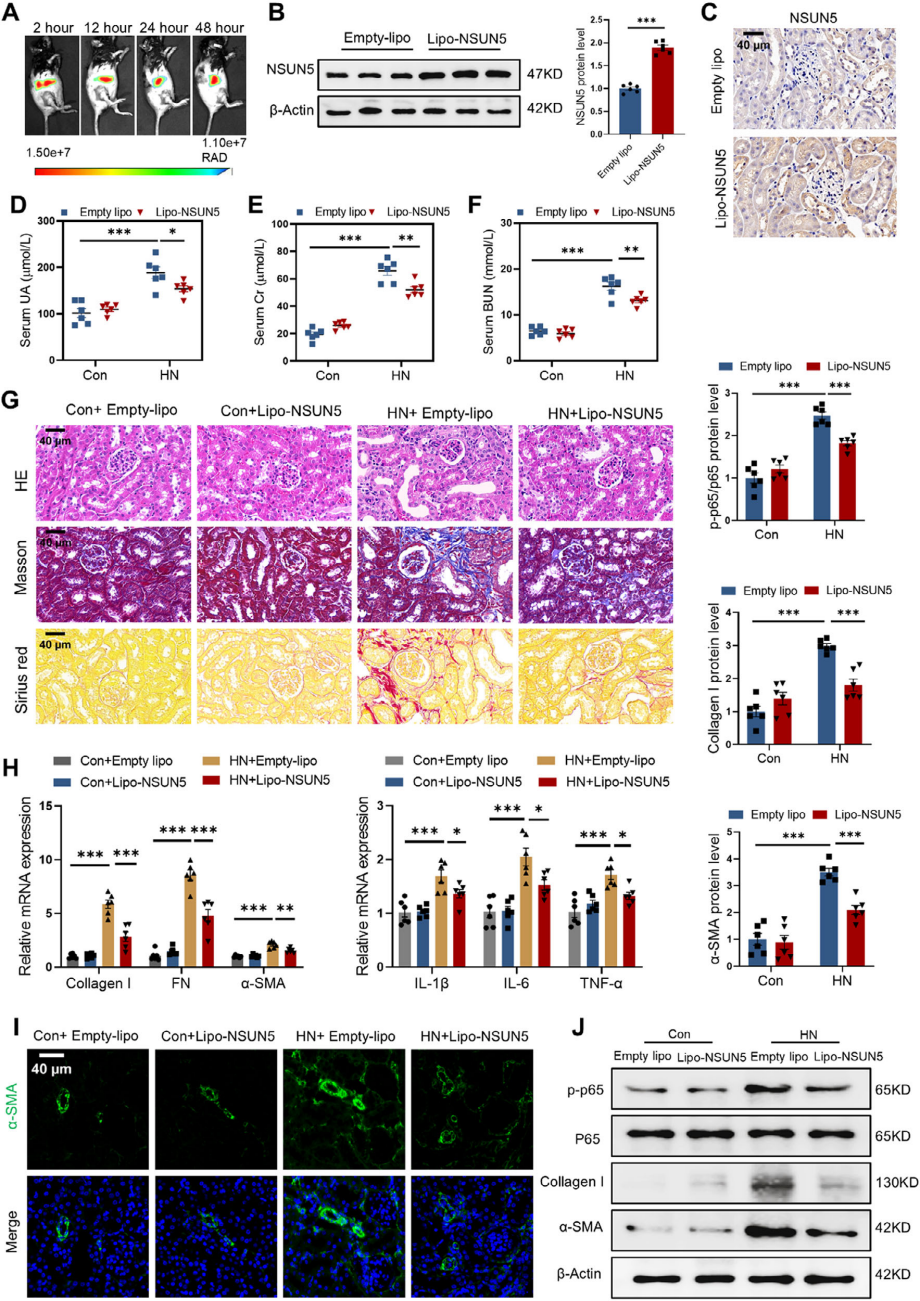
**Exogenous NSUN5 administration protects against renal injury in HN mice**. (A) In vivo imaging of mice at different time points after tail‐vein injection of Cy7‐label liposomes. (B,C) Western blot and IHC analyses of NSUN5 expression (n = 6). (D–F) Serum UA, Cr, and BUN levels (n = 6). (G) Representative images of H&E, Masson's trichrome, and Sirius Red staining (n = 3). (H) Real‐time PCR analysis of IL‐1β, IL‐6, TNF‐α, α‐SMA, collagen‐I, and FN (n = 6). (I) IF staining of α‐SMA. (J) Western blot analysis of p‐p65, collagen I, and α‐SMA (n = 6). Data are presented as the mean ± SEM. *P* values were calculated using one‐way ANOVA with Tukey's post hoc test, ^*^ *p* < 0.05, ^**^ *p* < 0.01, ^***^ *p* < 0.001.

To explore the therapeutic potential of NSUN5 in HN, we produced recombinant NSUN5 and encapsulated it in kidney‐targeting liposomes. Mice received tail vein injections of empty liposomes (Empty‐lipo) or NSUN5‐loaded liposomes (Lipo‐NSUN5) (Figure S9A). In vivo whole‐animal imaging showed that Cy7‐label liposomes preferentially accumulated in the kidneys, with a strong fluorescent signal detectable up to 48 h post‐injection (Figure 9A; Figure S9B); therefore, injections were administered once every other day. Western blot and IHC analyses confirmed a marked increase in renal NSUN5 protein levels in the Lipo‐NSUN5 group (Figure 9B,C). Functionally, NSUN5 supplementation significantly reduced serum UA, Cr, and BUN levels in HN mice (Figure 9D–F). Consistent with our previous findings, HE, Masson's trichrome, Sirius Red staining and gross morphology of the kidneys revealed that Lipo‐NSUN5 treatment markedly attenuated renal tissue damage and collagen deposition (Figure 9G; Figure S9C). Moreover, mRNA levels of fibrotic markers (collagen I, fibronectin and α‐SMA) and pro‐inflammatory cytokines (IL‐1β, IL‐6 and TNF‐α) were substantially downregulated (Figure 9H). IF analysis showed reduced renal α‐SMA expression (Figure 9I). Western blot analysis further demonstrated that recombinant NSUN5 effectively suppressed collagen I and α‐SMA expression, inhibited p65 phosphorylation, and upregulated ABCG2 expression (Figure 9J; Figure S9D). Collectively, these results indicate that exogenous NSUN5 administration substantially alleviates renal inflammation and fibrosis in HN mice, highlighting its potential as a therapeutic strategy for HN.

## 3 Discussion

Hyperuricaemia is a significant risk factor for the onset and progression of chronic kidney disease (CKD), with a prevalence as high as 36.6%–50% among patients with CKD in China; this proportion increases further as renal function deteriorates [22]. Although previous studies have revealed various mechanisms underlying HN, including abnormal activation of the renin–angiotensin system and oxidative stress [23], the role of RNA epigenetic modifications in HN remains poorly understood. This study demonstrates that the m5C methyltransferase NSUN5 is downregulated in HN, and both in vivo and in vitro experiments confirm that its overexpression significantly alleviates renal inflammation, fibrosis, and ferroptosis. Mechanistically, we uncover a novel signalling axis whereby NSUN5 enhances m5C modification and stabilisation of SCD1 mRNA in a YBX2‐dependent manner, thereby suppressing ferroptosis, attenuating NF‐κB–mediated inflammation, and promoting ABCG2‐dependent UA excretion, ultimately exerting multifaceted renoprotective effects.

The function of RNA m5C modification depends on its recognition by reader proteins, and previous studies suggest that it can enhance RNA stability [24, 25, 26]. This study identifies SCD1 as a key downstream target of NSUN5‐mediated m5C modification. Further experiments demonstrate that NSUN5 overexpression significantly increases m5C enrichment on SCD1 mRNA and prolongs its half‐life. Regarding the recognition mechanism, we show that YBX2 acts as an m5C reader and directly binds to SCD1 mRNA to maintain its stability under UA stimulation. The YBX family (YBX1, YBX2, and YBX3) plays a critical role in mRNA stability and translation [18]. Notably, YBX2, a homologue of YBX1, was recently confirmed as a novel RNA m5C‐binding protein [27]. This study further expands our understanding of YBX2 function in kidney disease, suggesting its potential involvement in the pathological process of HN through recognition of m5C modification on SCD1 mRNA.

SCD1, a key desaturase in lipid metabolism, has recently been found to play important roles in regulating inflammation [28] and cell death [29]. This study demonstrates that SCD1 activation significantly inhibits the phosphorylation and nuclear translocation of NF‐κB p65, thereby downregulating the expression of pro‐inflammatory factors. This finding is consistent with that of previous research, which reported that monounsaturated fatty acids produced by SCD1 catalysis possess anti‐inflammatory activity and can suppress activation of the NF‐κB pathway [20]. Furthermore, we show that upregulation of SCD1 promotes the expression of the urate transporter ABCG2, consistent with our prior research showing that NF‐κB inhibition upregulates ABCG2 to enhance UA excretion [21]. These results suggest that the NSUN5–SCD1 axis may exert renoprotective effects through a dual mechanism: suppressing the inflammatory response and enhancing UA excretion capacity. Notably, recent studies have shown that SCD1 is also involved in regulating pyroptosis [30]. In this study, the NSUN5–SCD1 axis suppresses NF‐κB activity and the production of mature pyroptosis‐related cytokines such as IL‐1β. Although this study focuses on ferroptosis, NF‐κB is also a key upstream regulator of pyroptosis. Therefore, we speculate that the NSUN5–SCD1 axis may indirectly alleviate hyperuricaemia‐induced renal tubular epithelial cell pyroptosis by inhibiting NF‐κB, offering a new direction for future research.

Ferroptosis is a form of non‐apoptotic cell death driven by iron‐dependent lipid peroxidation [31, 32] that can induce renal cell death, leading to impaired renal function [33, 34, 35]. Recent studies have found that the ferroptosis inhibitor Ferrostatin‐1 exhibits both UA‐lowering and renoprotective effects [36], suggesting a potential role for ferroptosis in HN progression. SCD1, a lipid‐modifying enzyme, has been identified as a key regulator of ferroptosis in tumour cells [37, 38, 39, 40]. However, its involvement in ferroptosis regulation in HN remains unclear. By integrating BS‐seq and RNA‐seq, we show that genes differentially methylated by NSUN5 are significantly enriched in the ferroptosis pathway. Electron microscopy and molecular marker analyses confirm the occurrence of ferroptosis in HN and identify the NSUN5–SCD1 axis as a key negative regulatory pathway. These findings provide a potential therapeutic avenue for HN by targeting this axis to suppress ferroptosis in renal tubular cells.

Finally, to investigate the therapeutic potential of NSUN5, recombinant NSUN5‐loaded liposomes were used. Exogenous administration of these liposomes in an HN mouse model effectively recapitulates the protective effects of genetic overexpression, significantly improving renal function and alleviating histopathological damage, underscoring its clinical potential.

The limitation of this study is that the precise molecular mechanisms by which SCD1 precisely regulates NF‐κB activity require further investigation.

In summary, this study systematically elucidates the protective role of NSUN5 through m5C modification in HN. We demonstrate that NSUN5, by enhancing YBX2‐dependent SCD1 mRNA stability, promotes UA excretion and inhibits inflammation via the SCD1–NF‐κB–ABCG2 axis, while concurrently protecting renal tubular cells by suppressing ferroptosis. These findings not only advance the understanding of HN pathogenesis but also suggest that targeting the NSUN5/YBX2/SCD1 axis represents a promising therapeutic strategy for HN.

## 4 Experimental Section

### 4.1 Reagents

UA (U0881, Sigma–Aldrich, Switzerland); potassium oxonate (P831461, Macklin, Shanghai, China); adenine (A8626, Sigma–Aldrich, China); TO901317 (HY‐10626, MedChemExpress, USA); serum Cr, BUN, and UA assay kits (Jiancheng Bioengineering Institute, Nanjing, China); modified Masson's trichrome staining kit (Besso Biotechnology Institute, Wuhan, China); H&E staining kit (Beyotime Biotechnology, Jiangsu, China); and Sirius Red staining kit (Sbjbio, Nanjing, China). Detailed information on the primary antibodies used is provided in Table S1.

### 4.2 HN Mouse Model Construction and Treatment

All animal experiments were conducted in strict accordance with the *Guidelines for the Care and Use of Laboratory Animals*. Male C57BL/6J mice (20–25 g), aged 6–8 weeks, were obtained from the Laboratory Animal Center of Anhui Medical University. The experimental protocol was approved by the Animal Experiment Ethics Committee of Anhui Medical University (No. LLSC20241177). After one week of acclimatisation, the mice were randomly assigned to control and HN groups (n = 6 per group). To establish the HN model, mice received daily oral gavage of a suspension containing potassium oxonate (2100 mg/kg) and adenine (140 mg/kg) in 0.5% sodium carboxymethyl cellulose (CMC‐Na). Control mice received an equal volume of 0.5% CMC‐Na solution daily. After 21 days, the mice were anaesthetized via intraperitoneal injection of pentobarbital sodium (200 mg/kg) for terminal sample collection.

### 4.3 Generation and Treatment of G9KO Mice

GLUT9 is a urate transporter encoded by *SLC2A9*. Previous studies have demonstrated that global G9KO mice exhibit significantly elevated UA levels and early‐onset nephropathy, with renal inflammatory responses and fibrosis extending to most regions of the cortex by 6 weeks, confirming their utility in research on hyperuricaemia‐associated renal disease [41]. GLUT9 knockout mice (C57BL/6J background, generated by Cyagen Biosciences, Suzhou) were randomly assigned to four groups (n = 6 per group): WT, WT + NSUN5 OE, G9KO, and G9KO + NSUN5 OE.

### 4.4 Generation and Administration of AAV9

AAV9 was synthesized by Shanghai Hanheng Biotechnology. Male C57BL/6J mice were randomly divided into four groups (n = 6 per group): AAV9‐EV, AAV9‐NSUN5 OE, AAV9‐EV‐HN, and AAV9‐NSUN5 OE‐HN. Using an insulin syringe, 100 µL of virus at a titre of 1 × 10^12^ was injected via the tail vein. After 4 weeks, HN modelling was performed.

### 4.5 Generation and Treatment of NSUN5^tecKI^ Mice

The renal tubule‐specific NSUN5 overexpression mice (C57BL/6J background; IHM, China) were generated by the Hefei Institute of Physical Science. In brief, CAG‐LSL‐NSUN5 mice (NSUN5^flox/flox^) were constructed using transgenic technology and crossed with *Cdh16*‐Cre mice to generate the renal tubule‐specific NSUN5 overexpression mice (*Cdh16*‐Cre+*NSUN5* ^flox/flox^, referred to as NSUN5^tecKI^). Homozygous floxed mice lacking Cre expression were designated as NSUN5^fl/fl^. The mice were divided into four groups (n = 6 per group): NSUN5^fl/fl^, NSUN5^tecKI^, HN + NSUN5^fl/fl^, and HN + NSUN5^tecKI^.

### 4.6 Western Blotting

Kidney tissues or cells were lysed on ice in RIPA buffer, and the resulting supernatant was collected for protein extraction. Proteins were separated by SDS‐PAGE and transferred onto PVDF membranes. The membranes were blocked with 5% non‐fat milk at room temperature for 1 h, followed by incubation with diluted primary antibodies overnight at 4°C. The membranes were then developed using an ECL reagent (BL520A, BioSharp, China), and band intensities were quantified using ImageJ software.

### 4.7 Real‐Time PCR

Total RNA was extracted from cells or tissues using the NcmSpin Cell/Tissue Total RNA Kit (M5105, NCM Biotech, China), according to the manufacturer's instructions. Real‐time PCR was performed on a CFX96 system using the SYBR Green SuperMix. Primer sequences used are listed in Table S3.

### 4.8 IF

Cells or tissue sections were fixed with 4% paraformaldehyde, permeabilized with 0.1% Triton X‐100, and blocked with 5% BSA to prevent nonspecific binding. After overnight incubation with primary antibodies at 4°C, the samples were treated with fluorescent secondary antibodies for 1 h at room temperature in the dark, followed by washing with PBS for 5 min. Nuclei were counterstained with DAPI for 10 min and observed under a fluorescence microscope (Zeiss Spot, Carl Zeiss Microscopy GmbH, Göttingen, Germany).

### 4.9 IHC

After dewaxing and rehydration, tissue sections were subjected to antigen retrieval. Sections were then incubated with 3% H_2_O_2_ at room temperature for 10 min, followed by blocking with 5% BSA for 30 min. The sections were incubated with the primary antibody overnight at 4°C. Subsequently, the sections were incubated with HRP‐conjugated secondary antibody at room temperature for 1 h, followed by DAB staining. Finally, nuclei were counterstained with haematoxylin.

### 4.10 Cell Lines and Culture Conditions

mTECs were provided by Professor Hui Yao Lan at The Chinese University of Hong Kong. mTECs were cultured in Dulbecco's modified Eagle's medium/nutrient mixture F‐12 (DMEM/F12) (C11330500BT, Gibco) supplemented with 10% foetal bovine serum (FBS) in an atmosphere of 95% air and 5% CO_2_. After 12 h of serum starvation in DMEM/F12 medium containing 0.5% FBS, cells were treated with UA (15 mg/dL) for 24 h to establish the in vitro model.

### 4.11 Cell Transfection

Plasmid and siRNA transfections were performed using Lipofectamine 3000 (Invitrogen) according to the manufacturer's instructions. The medium was changed after 6 h, and samples were collected for analysis 48–72 h later. Plasmids were obtained from GenePharma (Shanghai, China), and siRNAs were purchased from Tsingke Biotech (Nanjing, China). Detailed information on the synthesized siRNAs is provided in Table S2.

### 4.12 BS‐seq and RNA‐seq

BS‐seq and RNA‐seq analyses were performed by Wuhan Kangce Technology Co., Ltd.

### 4.13 TEM

Tissue samples from mouse kidneys or cultured cells were fixed in ice‐cold 2.5% glutaraldehyde at 4°C for 2 h. The samples were then processed using standard procedures, including dehydration, infiltration, embedding, sectioning, and staining. Finally, samples were examined under a Hitachi transmission electron microscope (HT7800, Japan) at magnifications of 6000× and 15 000×.

### 4.14 RNA Stability

To evaluate SCD1 mRNA stability, cells were treated with actinomycin D (5 µg/mL; GC16866, GLPBIO) to inhibit transcription. Samples were collected at 0, 3, and 6 h after treatment. Total RNA was extracted, and SCD1 mRNA levels were quantified using real‐time PCR.

### 4.15 RIP and MeRIP

The RIP assay was performed using the PureBinding Co‐Immunoprecipitation Kit (P0101, GENESEED, Guangzhou, China) following the manufacturer's protocol. Briefly, approximately 1 × 10^7^ mTECs were collected and lysed. These lysates were incubated with YBX2 antibody–conjugated magnetic beads at 4°C with rotation (10 rpm) for 2 h to overnight, with mouse IgG used as the negative control. The antibody–bead complexes were then washed 4–6 times, followed by RNA extraction. The interaction between SCD1 mRNA and YBX2 was analysed using real‐time PCR. For MeRIP, total RNA was extracted and fragmented. The anti‐m5C antibody (68301‐1‐Ig, Proteintech) was incubated with pre‐treated Protein A/G beads at 4°C for 2 h. After washing, purified mRNA was mixed with the antibody–bead complexes and incubated overnight at 4°C. Finally, RNA bound to the magnetic beads was eluted and subjected to RNA extraction.

### 4.16 ROS Detection

ROS levels were measured using the ROS Assay Kit (S0033S, Beyotime Biotechnology, China) according to the manufacturer's instructions. Images were randomly acquired using the ImageXpress Micro Confocal Microscope (Molecular Devices, USA).

### 4.17 Preparation of Recombinant NSUN5

To establish an expression system for NSUN5 production, the mouse *NSUN5* gene (NM_145414.2) was cloned into the pET‐28a vector. The target fragment was amplified using PCR, gel purified, and inserted into the linearized vector using Gibson assembly. The resulting construct was transformed into chemically competent BL21(DE3) cells, plated, and positive clones were selected. Recombinant plasmids were confirmed using colony PCR, restriction digestion with XbaI, and DNA sequencing. Verified transformants were cultured in LB medium at 37°C to an OD_600_ of 0.6–0.8, induced with 0.4 mmol/L IPTG, and expressed overnight. Cells were harvested and lysed, and protein expression was verified via western blotting using an anti‐His antibody. The protein was purified under denaturing conditions on an IDA–nickel column, refolded with renaturation buffer, concentrated, and yielded 0.5 g/L of recombinant NSUN5 with 85 % purity.

### 4.18 Preparation of Liposomes

Liposomes were prepared by thin‐film hydration followed by sonication. Phospholipids (60 mg), cholesterol (20 mg), and DSPE‐PEG (5 mg) were dissolved in chloroform/methanol (12/4 mL). The organic solvents were removed by rotary evaporation at 40°C for 30 min to form a lipid film, which was further dried in a 40°C oven for 30 min. To hydrate the film, 10 mL of aqueous buffer was added, and the mixture was incubated at 37°C with shaking for 30 min, followed by sonication at 200 W for 10 min. The resulting liposomal suspension was filtered and subjected to size analysis. For NSUN5‐loaded liposomes, 3 mg of DSPE‐PEG‐NH_2_ was included in the above formulation, and the hydration step was performed using the protein solution instead of water. Cy7‐label liposomes were prepared by incubating 2 mL of liposome suspension with 40 µL of Cy7 in the dark with stirring for 3 h. Free dye was removed by dialysis against 5 % glucose for 24 h with 2–3 buffer changes.

### 4.19 Statistical Analysis

Data are presented as the mean ± SEM. An unpaired two‐tailed Student's *t*‐test was used to compare the means of two groups. Multiple comparisons were performed using one‐way ANOVA followed by Tukey's post hoc test. A *p*‐value < 0.05 was considered statistically significant. GraphPad Prism 8 software was used for all analyses.

## Author Contributions

J.J., X.M.M., and F.H.L. provided overall supervision and were responsible for data curation, resources, software development, visualization, and validation. X.X.S. and X.G.S. contributed to data curation, developed the methodology, and prepared the original draft. Y.Y., K.Z., and C.A.L. contributed to the methodology, while J.W., H.X.X., S.Y.N., D.X.L., and Z.H.H. were responsible for both methodology and investigation.

## Funding

This work was supported by the National Natural Science Foundation of China (no.82473992)

## Ethics Statement

The experimental protocol was approved by the Animal Experiment Ethics Committee of Anhui Medical University (No. LLSC20241177).

## Conflicts of Interest

The authors declare no conflicts of interest.

## Supporting information




**Supporting File 1**: advs75092‐sup‐0001‐SuppMat.docx.

**Supporting File 2**: advs75092‐sup‐0002‐DataFile.zip.

## Data Availability

The data that support the findings of this study are available from the corresponding author upon reasonable request.
