# Supplementary material for: NSUN5 Attenuates Renal Injury and Ferroptosis in Hyperuricaemic Nephropathy Through YBX2‐Dependent Stabilisation of SCD1 m5C Methylation
Source: Adv Sci (Weinh). 2026 Apr 21;13(34):e21459. doi: 10.1002/advs.202521459 (PMC13285163; doi:10.1002/advs.202521459)
Supplement: Supplementary file 1 — Supporting File 1: advs75092‐sup‐0001‐SuppMat.docx. [file ADVS-13-e21459-s001.docx]

Supporting Information

**NSUN5 Attenuates Renal Injury and Ferroptosis in Hyperuricaemic Nephropathy through YBX2-Dependent Stabilisation of SCD1 m5C Methylation**

*Xiu-xiu Song, Xiao-guo Suo, Yue Yu, Kuo Zhang, Chen-ao Li, Jie Wang, Hui-xia Xu, Si-yu Niu, Dong-xue Lv, Zi-hao He, Feng-he Li*, Xiao-ming Meng*, and Juan Jin**


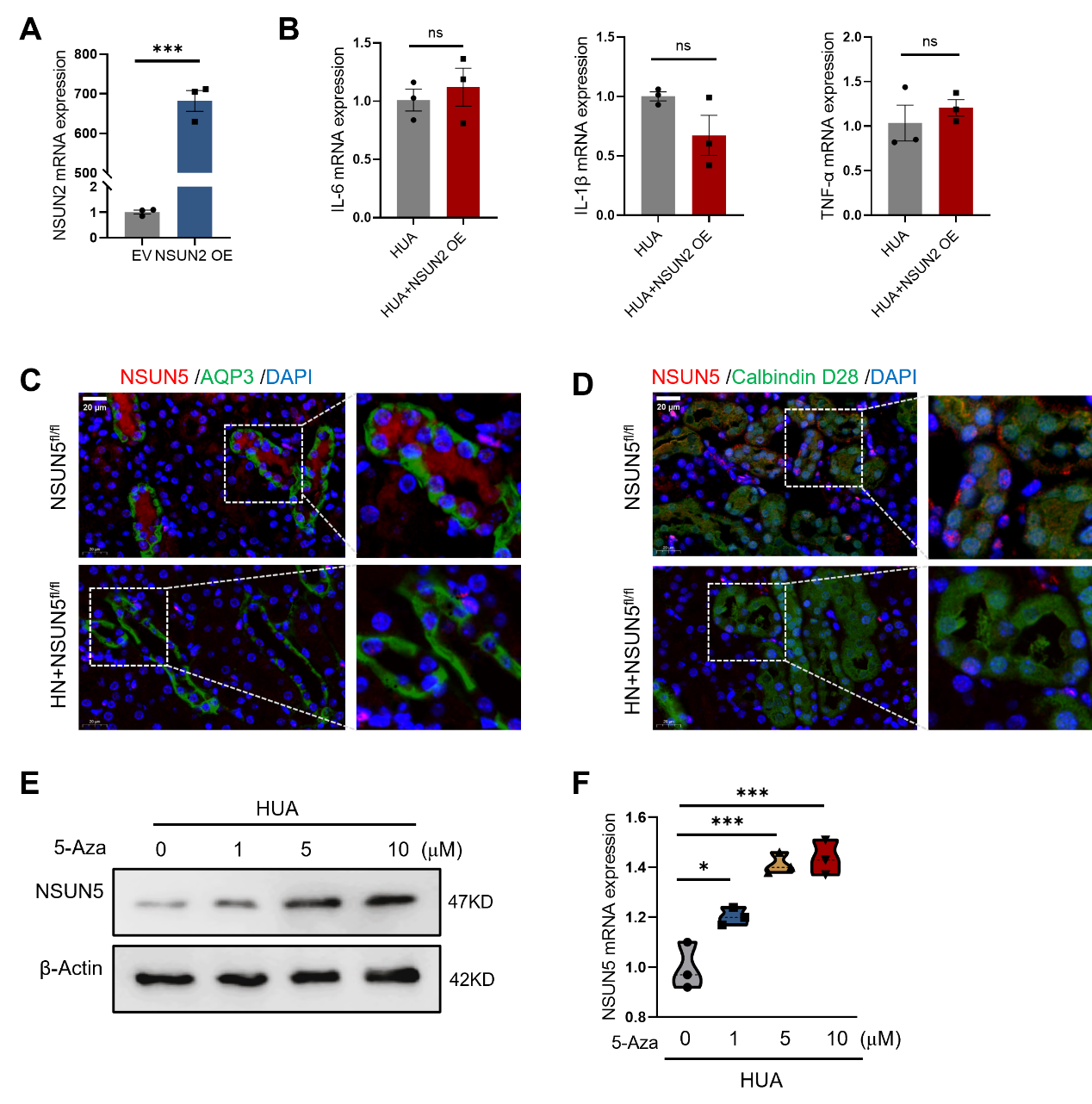


**Figure S1.** Reduced NSUN5 expression in *in vivo* and *in vitro* HN models. A) Real-time PCR analysis of NSUN2 overexpression efficiency in mTECs (n = 3). B) Real-time PCR analysis showing NSUN2 overexpression did not reduce UA-induced inflammation in mTECs (n = 3). C-D) Co-localisation of NSUN5 with aquaporin 3 or calbindin D28. E-F) 5-Aza (a DNA methyltransferase inhibitor) upregulates both protein expression and mRNA level of NSUN5 in a dose-dependent manner (n = 3). Data are presented as the mean ± SEM. *P* values were calculated using one-way ANOVA with Tukey’s post hoc test, **P* < 0.05, ***P* < 0.01, ****P* < 0.001.

**
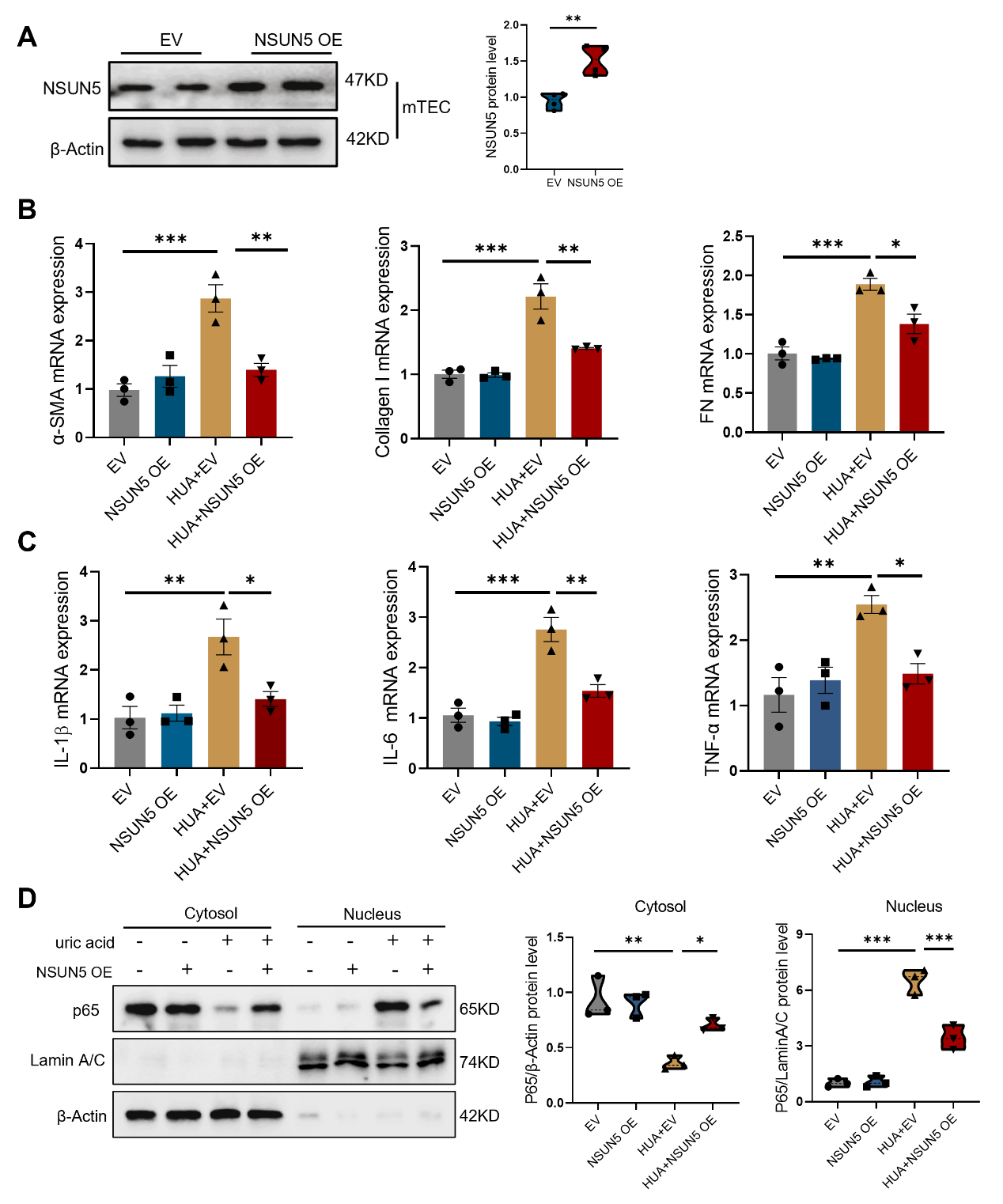
**

**Figure S2.** NSUN5 overexpression suppresses UA-induced fibrosis and inflammation in mTECs. A) Western blot analysis confirming successful overexpression of NSUN5 (n = 3). B-C) NSUN5 overexpression attenuates UA-induced increases in the mRNA levels of fibrotic markers (α-SMA, collagen I, FN), as well as inflammatory cytokines (IL-1β, IL-6, TNF-α) (n = 3). D) Western blot analysis of cytoplasmic and nuclear p65 protein expression, with β-Actin and Lamin A/C serving as loading controls (n = 3). Data are presented as the mean ± SEM. *P*-values were calculated using one-way ANOVA with Tukey’s post hoc test, **P* < 0.05, ***P* < 0.01, ****P* < 0.001.


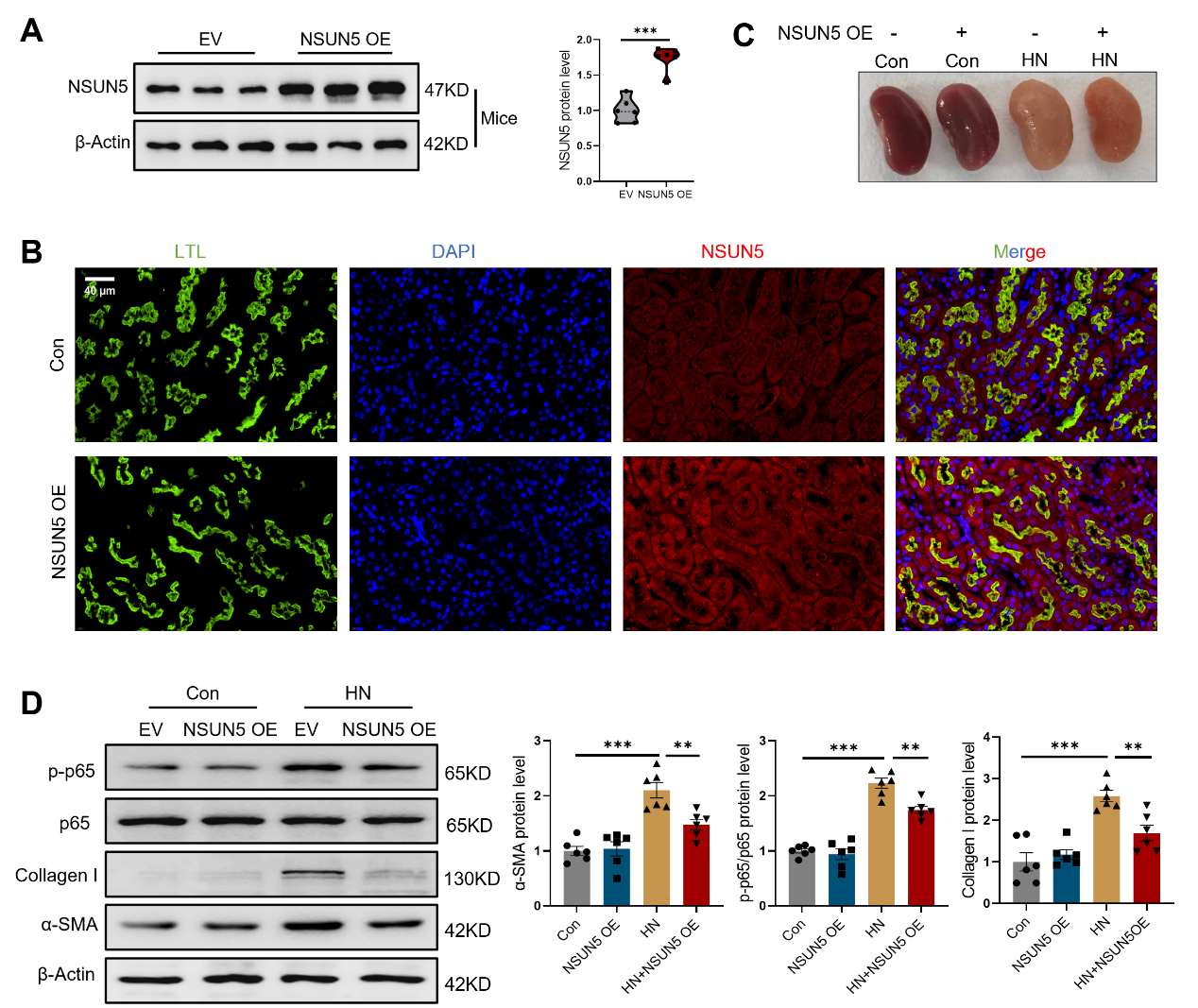


**Figure S3.** NSUN5 overexpression ameliorates renal injury in HN mice. A–B) Western blot and IF analyses evaluating the efficiency of AAV9-NSUN5 overexpression. C) Representative gross morphology of kidneys. D) Western blot analysis and quantification of p-p65, collagen I, and α-SMA protein levels (n = 6). Data are presented as the mean ± SEM. *P* values were calculated using one-way ANOVA with Tukey’s post hoc test, **P* < 0.05, ***P* < 0.01, ****P* < 0.001.


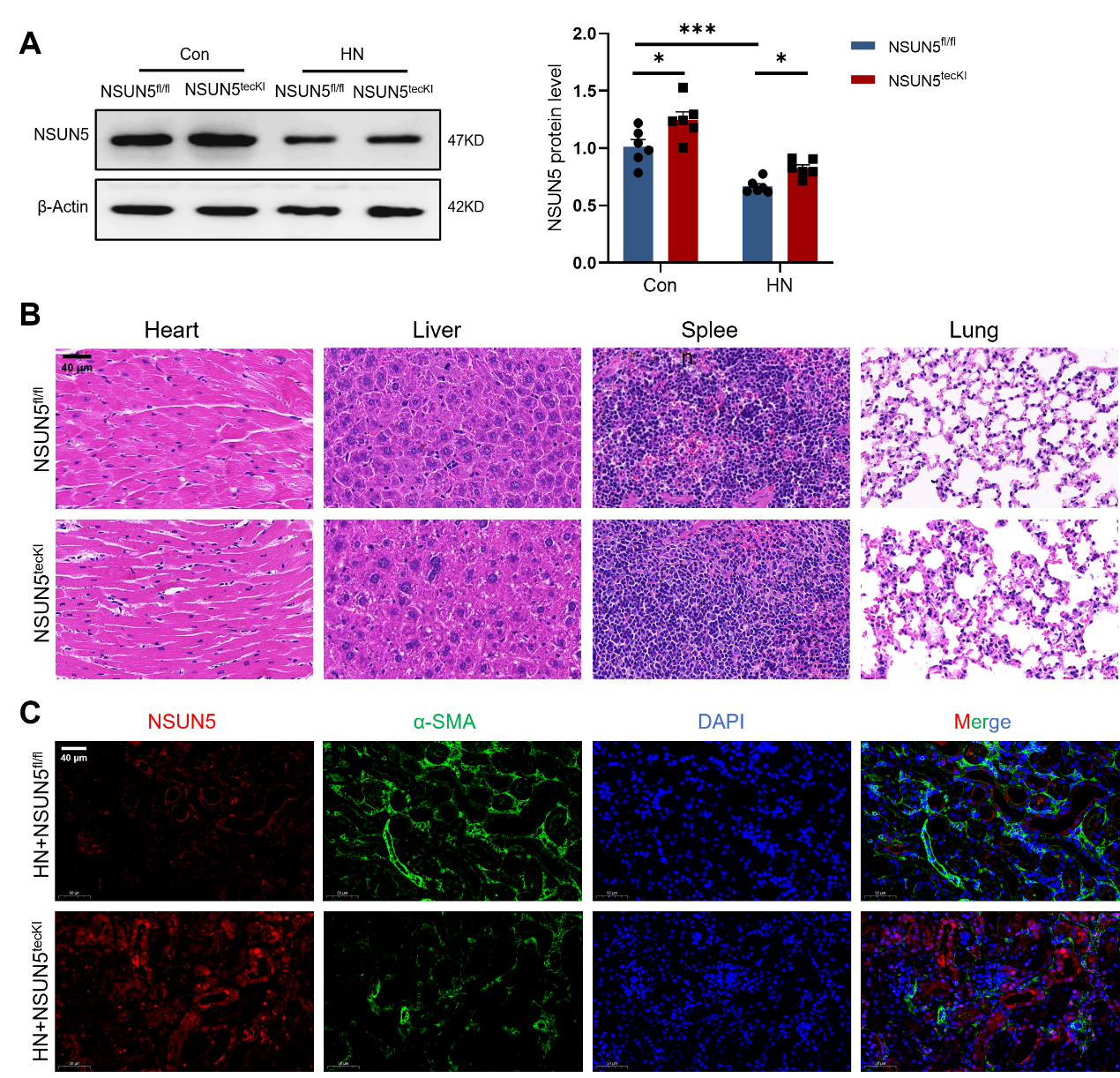


**Figure S4.** Tubule-specific overexpression of NSUN5 exerts a protective effect against renal injury in HN mice. A) Western blot analysis and quantification of NSUN5 protein expression in HN mice (n = 6). B) Representative H&E staining images of the heart, liver, spleen, and lung. C) IF co-staining of NSUN5 (red) and α-SMA (green). Data are presented as the mean ± SEM. *P* values were calculated using one-way ANOVA with Tukey’s post hoc test, **P* < 0.05, ***P* < 0.01, ****P* < 0.001.


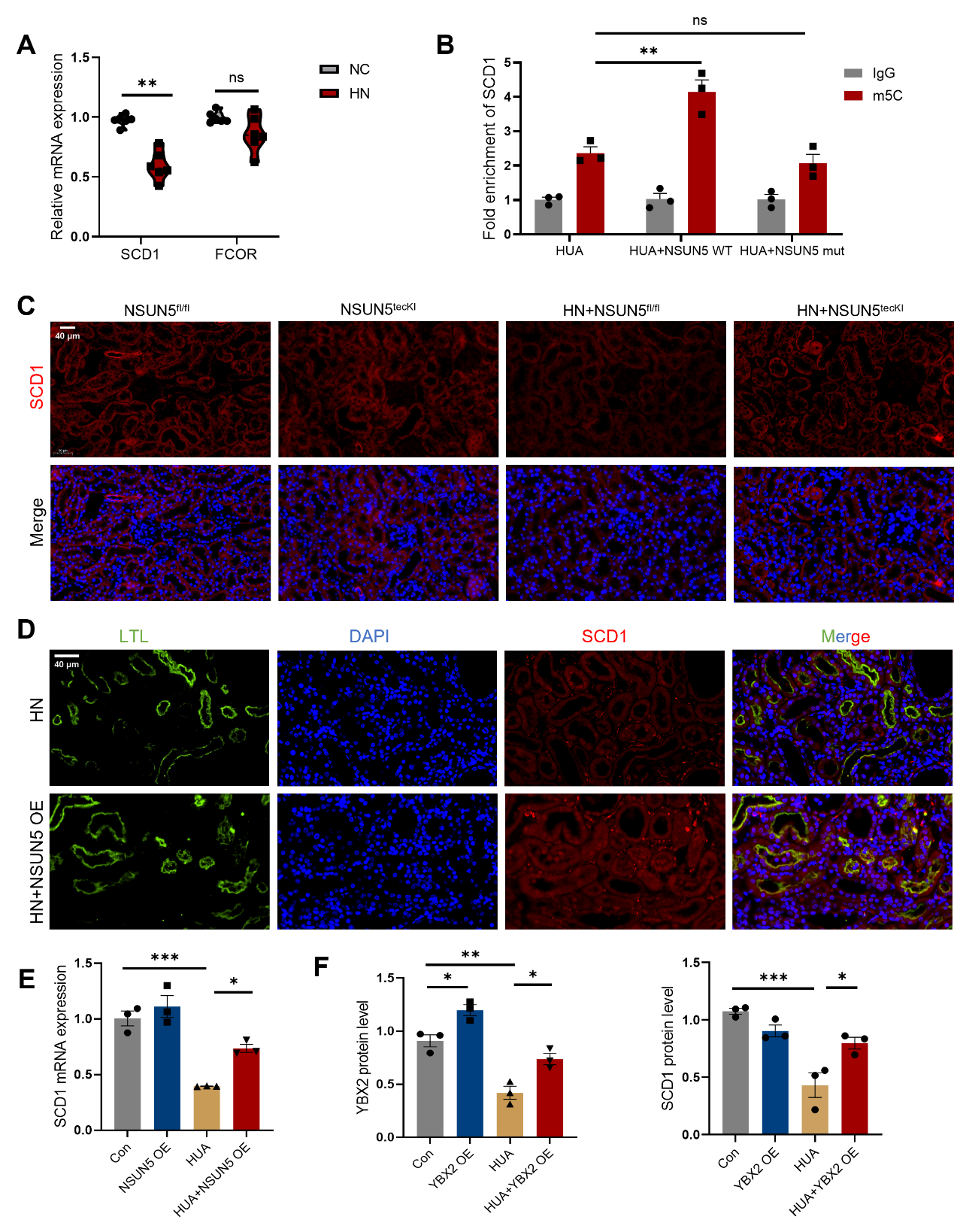


**Figure S5.** NSUN5 regulates SCD1 mRNA in an m5C–YBX2-dependent manner. A) Real-time PCR analysis of SCD1 and FCOR mRNA level (n = 6). B) MeRIP-qPCR analysis of changes in m5C modification of SCD1 in mTECs transfected with wild-type or mutant NSUN5 (n = 3). C) IF analysis showing that SCD1 expression is markedly reduced in HN + NSUN5^fl/fl^ mice, whereas tubule-specific NSUN5 overexpression restores SCD1 protein levels. D) IF co-staining of SCD1 and LTL in HN mice reveals that SCD1 is predominantly expressed in mTECs. (E) Real-time PCR analysis showing that NSUN5 overexpression suppresses the UA-induced downregulation of SCD1 mRNA (n = 3). (F) Quantitative analysis of YBX2 and SCD1 protein levels (n = 3). Data are presented as the mean ± SEM. *P* values were calculated using one-way ANOVA with Tukey’s post hoc test, **P* < 0.05, ***P* < 0.01, ****P* < 0.001.


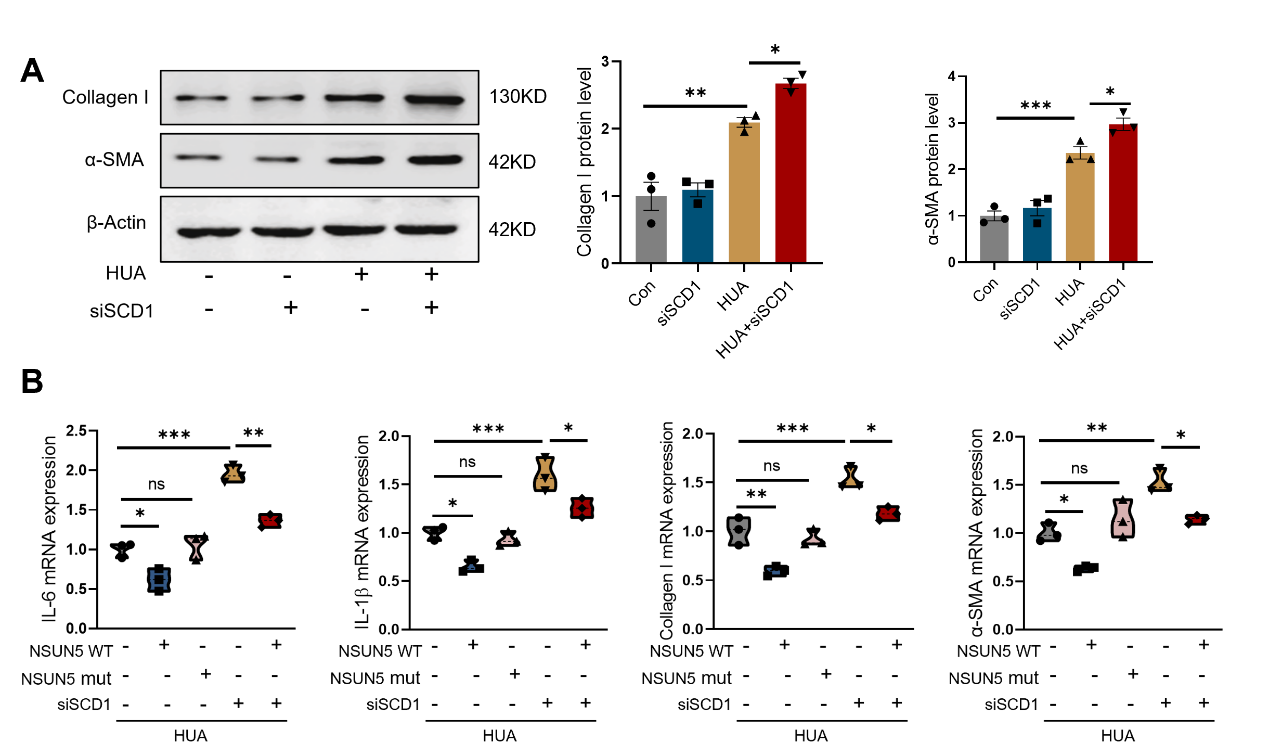


**Figure S6.** Silencing SCD1 promotes the expression of inflammatory and fibrotic factors in UA-induced mTECs. A) Western blot analysis of collagen I and α-SMA expression (n = 3). B) Real-time PCR analysis of IL-6, IL-1β, collagen I, and α-SMA mRNA level (n = 3). Data are presented as the mean ± SEM. *P* values were calculated using one-way ANOVA with Tukey’s post hoc test, **P* < 0.05, ***P* < 0.01, ****P* < 0.001.


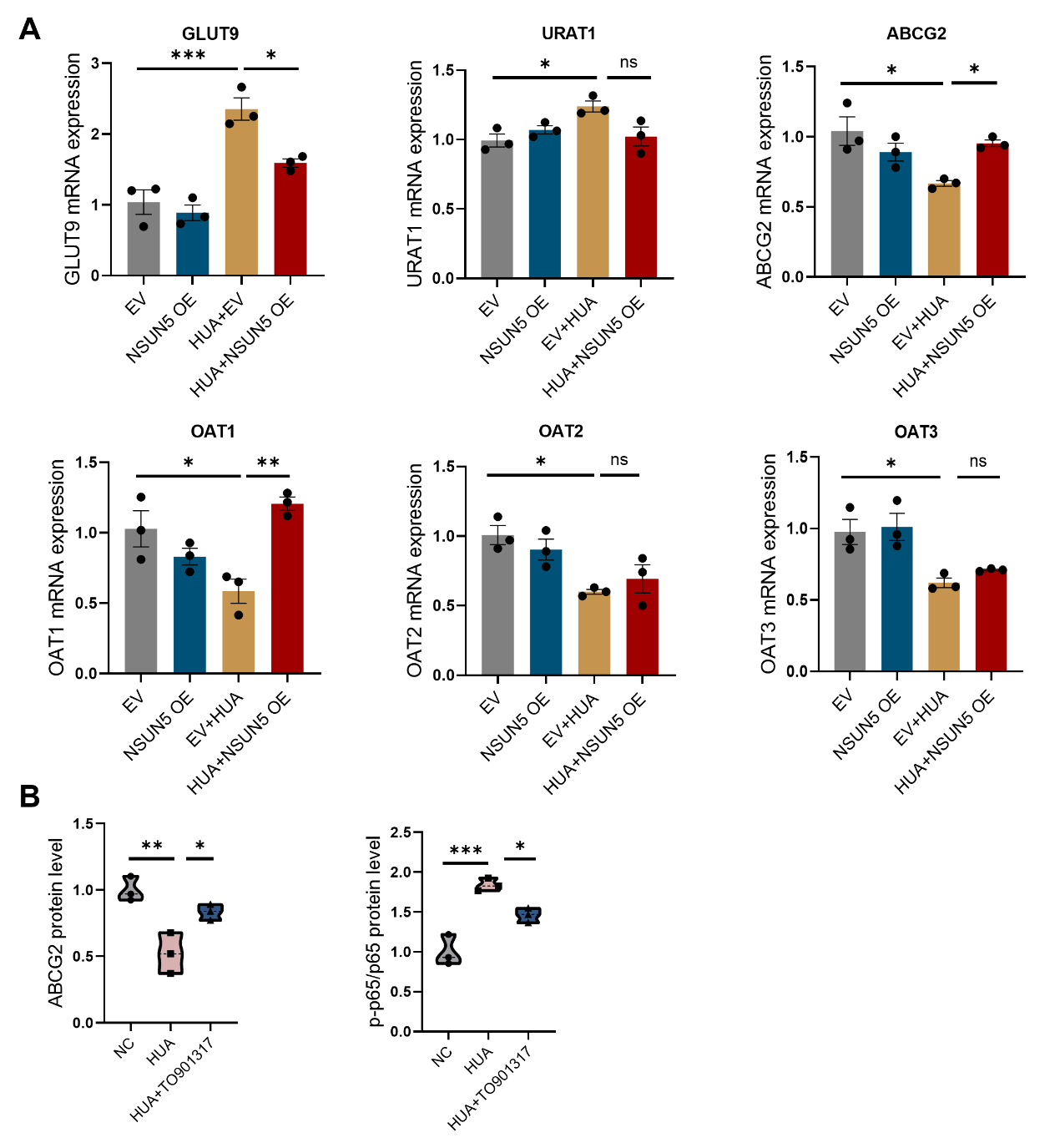


**Figure S7.** NSUN5 regulates the expression of UA transporters. A). Real-time PCR analysis the mRNA levels of GLUT9, URAT1, ABCG2, OAT1, OAT2, and OAT3 (n = 3). B) Quantification of ABCG2 and p-p65/p65 protein expression (n = 3). Data are presented as the mean ± SEM. *P* values were calculated using one-way ANOVA with Tukey’s post hoc test, **P* < 0.05, ***P* < 0.01, ****P* < 0.001.


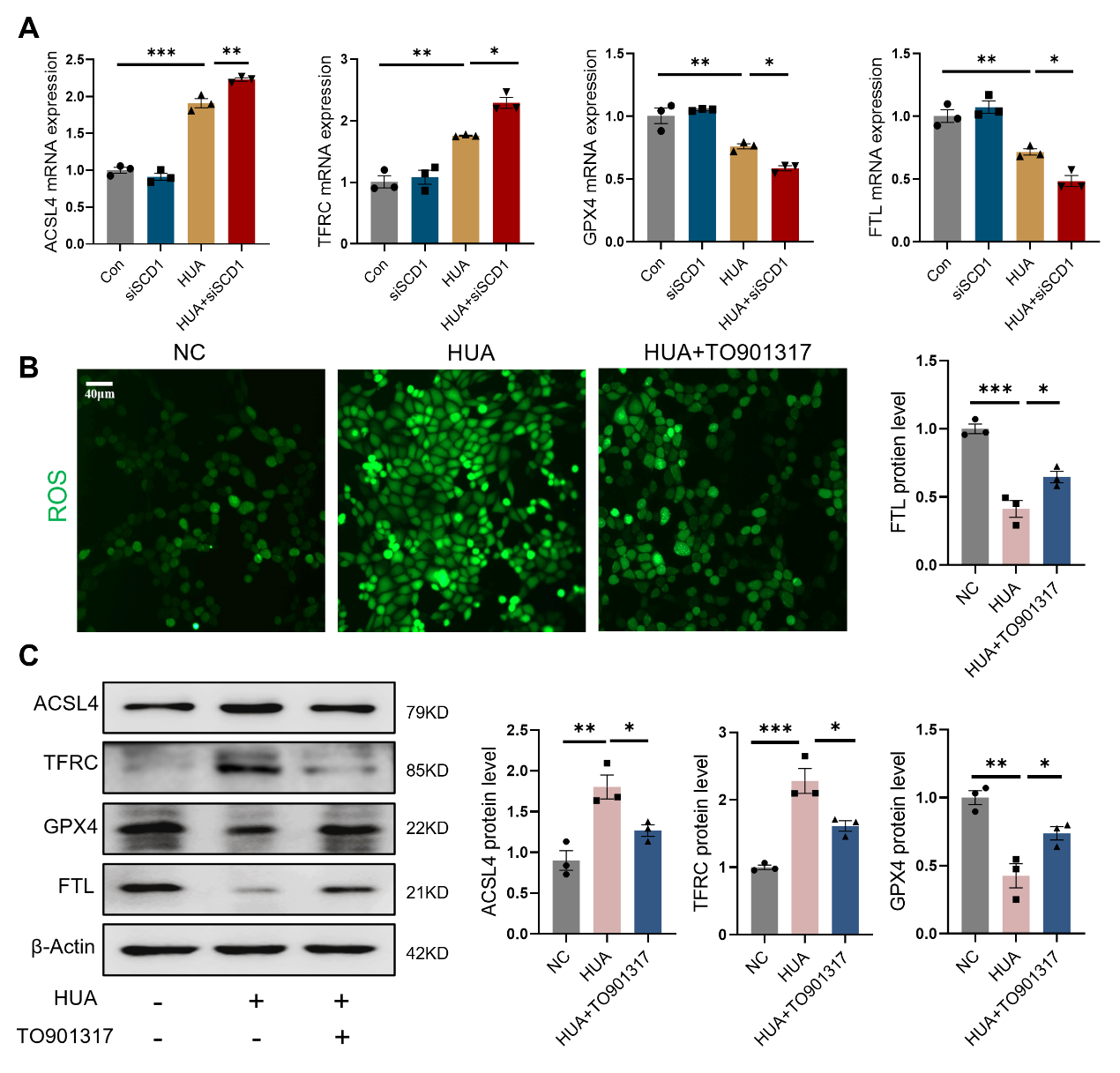


**Figure S8.** Elevated SCD1 suppresses UA-induced ferroptosis. A) Real-time PCR analysis showing that SCD1 silencing promotes the upregulation of pro-ferroptosis markers ACSL4 and TFRC and the downregulation of anti-ferroptosis markers GPX4 and FTL in UA-treated mTECs (n = 3). B) ROS staining revealing that TO901317 inhibits UA-induced ROS production in mTECs. C) Western blot analysis showing that TO901317 reverses UA-induced upregulation of pro-ferroptosis markers ACSL4 and TFRC and restores the expression of anti-ferroptosis proteins GPX4 and FTL in mTECs (n = 3). Data are presented as the mean ± SEM. *P* values were calculated using one-way ANOVA with Tukey’s post hoc test, **P* < 0.05, ***P* < 0.01, ****P* < 0.001.


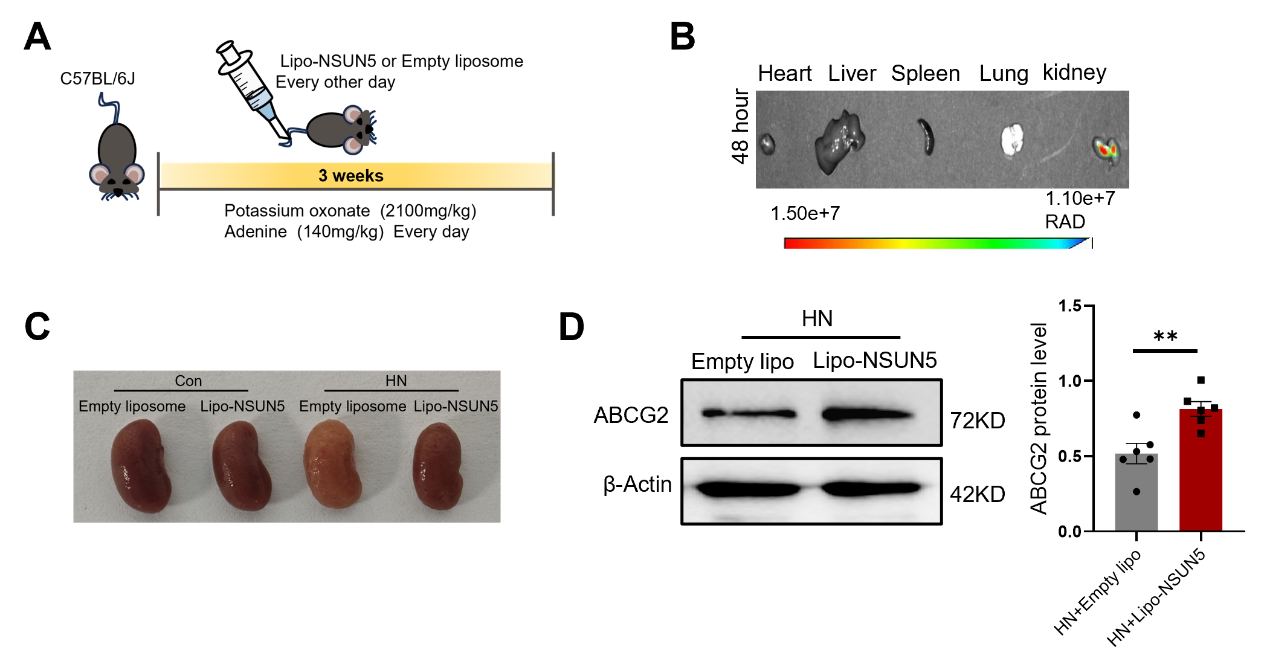


**Figure S9.** NSUN5 is a promising therapeutic target for HN. A) Schematic diagram of the experimental design. B) *In vivo* imaging of isolated mouse organs. C) Representative gross morphology of the kidneys. D) Western blot analysis of ABCG2 expression (n = 6). Data are presented as the mean ± SEM. *P* values were calculated using two-tailed unpaired Student's *t*-tests, **P* < 0.05, ***P* < 0.01, ****P* < 0.001.

**Supplementary Table S1.** Primary antibodies used in the experiments.

| **Name** | **Catalog Number** | **Company** |
| --- | --- | --- |
| Anti-α-SMA | ab124964 | Abcam |
| Anti-SCD1 | ab236868 | Abcam |
| Anti-SCD1 | ER1916-26 | HUABIO |
| Anti-Collagen I | bs-10423R | Bioss |
| Anti-GPX4 | bs-3884R | Bioss |
| Anti-β-Actin | bs-0061R | Bioss |
| Anti-ABCG2 | bs-0662R | Bioss |
| Lamin A/C | bsm-62887R | Bioss |
| Anti-NSUN5 | HA500544 | HUABIO |
| Anti-NSUN5 | 15449-1-AP | proteintech |
| Anti-m5C | 68301-1-Ig | proteintech |
| Anti- Phospho-NF-κB p65 | 82335-1-RR | proteintech |
| Anti- NF-κB p65 | D14E12 | Cell Signaling Technology |
| Anti-ACSL4 | R24265 | Zenbio |
| Anti-FTL | ET1610-78 | HUABIO |
| Anti-TFRC | ET1702-06 | HUABIO |
| Anti-YBX2 | sc-393840 | Santa Cruz |
| Anti-Aquaporin 3 | GB12533 | Servicebio |
| Anti-Calbindin | GB12268 | Servicebio |

**Supplementary Table S2.** The siRNA information of SCD1.

| **siRNA** | **Sense chain** | **Antisense chain** |
| --- | --- | --- |
| si-SCD1-1 | CGGUACAGUAUUCUUAUAA | UUAUAAGAAUACUGUACCG |
| si-SCD1-2 | CAUGAUAAAUGCAUAUACA | UGUAUAUGCAUUUAUCAUG |
| Si-SCD1-3 | GGCAGAGAUUUAAUAUUCU | AGAAUAUUAAAUCUCUGCC |

**Supplementary Table S3.** Primer sequences used in Real-time PCR analysis.

| **Genes** | **Species** | **Forward primer (5'-3')** | **Reverse primer (5'-3')** |
| --- | --- | --- | --- |
| β-Actin | Mouse | CATTGCTGACAGGATGCAGAA | ATGGTGCTAGGAGCCAGAGC |
| TNF-α | Mouse | CATCTTCTCAAAATTCGAGTGACAA | TGGAGTAGACAAGGTACAACCC |
| IL-1β | Mouse | GAAATGCCACCTTTTGACAGTG | GGATGCTCTCATCAGGACAG |
| IL-6 | Mouse | GAGGATACCACTCCCAACAGACC | AAGTGCATCATCGTTGTTCATACA |
| Collagen I | Mouse | TCCCTGGAATGAAGGGACAC | CTCTCCCTTAGGACCAGCAG |
| α-SMA | Mouse | GCACCTGGATCATTGCTTCC | TCCTTGGAAGTACTGCCGTT |
| FN | Mouse | TCACCTACGGAGAGACAGGA | TGTTGTTGATGGTGGCTGTG |
| SCD1 | Mouse | TTCTTGCGATACACTCTGGTGC | CGGGATTGAATGTTCTTGTCGT |
| ACSL4 | Mouse | CCT GAGGGGCTTGAAATTCAC | GTTGGTCTACTTGGAGGAACG |
| TFRC | Mouse | GTTTCTGCCAGCCCCTTATTAT | GCAAGGAAAGGATATGCAGCA |
| GPX4 | Mouse | TGTGCATCCCGCGATGATT | CCCTGTACTTATCCAGGCAGA |
| FTL | Mouse | CGTCAGAATTATTCCACCGAGG | GCCACGTCATCCCGATCAAA |
| YBX1 | Mouse | AAGGTCATCGCAACGAAGGTT | CAAATACGTCTTCCTTGGTGTCA |
| YBX2 | Mouse | CGGTGCTGGCAATCCAAGT | CCTTCCACGACATCAAACTCC |
| ALYREF | Mouse | ACCGAAACAACTTCCCGACAA | CACATCTGCTGTCCCTAAACTT |
| FMRP | Mouse | CAATGGCGCTTTCTACAAGGC | TCTGGTTGCCAGTTGTTTTCA |
| YTHDF2 | Mouse | GAGCAGAGACCAAAAGGTCAAG | CTGTGGGCTCAAGTAAGGTTC |
| RAD52 | Mouse | TCCACAGAGGGTGTGACAAC | CCATGCGGCTGCTAATGTACT |
| SRSF2 | Mouse | GCGCTCCAGATCAACCTCC | GCGCTCCAGATCAACCTCC |
| NSUN2 | Mouse | GTAAGTAGCGCTCACCTGCT | ATGCCAGGAAAGACCCACTG |
| NSUN3 | Mouse | ATGCCAGGAAAGACCCACTG | CGTCTCTCAGTTCCAGAGCC |
| NSUN4 | Mouse | GAGAGCACTGAGTGGACTTGA | GGCCAAAGATCCCCAAACTG |
| NSUN5 | Mouse | CTACCAGGTACCACGGTTCG | AGGCAGCAAGGGATCCAAAA |
| NSUN6 | Mouse | CCAAGCGTTAGGTGTGGTAGT | CGCCAAATAGGGCTTGGTGA |
| NSUN7 | Mouse | TCTGCCGCCAGTGATATTCC | GTGCTCAGCCGTCTTCACTA |
| TET1 | Mouse | GTCTGTATTCGCCCCTGGAC | TTGGTCACCTGAGTTTGGGG |
| TET2 | Mouse | GCTGTTCTCAGAATGAAACTAGAAA | TCTCTCTTCCTCTTTTGGCTCAG |
| TET3 | Mouse | GGGCAGGCAGCGTAGC | ATGAGGTGAGCCAATGGGTG |
