# Supplementary material for: NSUN5 Attenuates Renal Injury and Ferroptosis in Hyperuricaemic Nephropathy Through YBX2‐Dependent Stabilisation of SCD1 m5C Methylation
Source: Adv Sci (Weinh). 2026 Apr 21;13(34):e21459. doi: 10.1002/advs.202521459 (PMC13285163; doi:10.1002/advs.202521459)

**NSUN5 Attenuates Renal Injury and Ferroptosis in Hyperuricaemic Nephropathy through YBX2-Dependent Stabilisation of SCD1 m5C Methylation** *Xiu-xiu Song, Xiao-guo Suo, Yue Yu, Kuo Zhang, Chen-ao Li, Jie Wang, Hui-xia Xu, Si-yu Niu, Dong-xue Lv, Zi-hao He, Feng-he Li*, Xiao-ming Meng* Juan Jin**

**^SUPPLEMENTARY INFORMATION^**

| 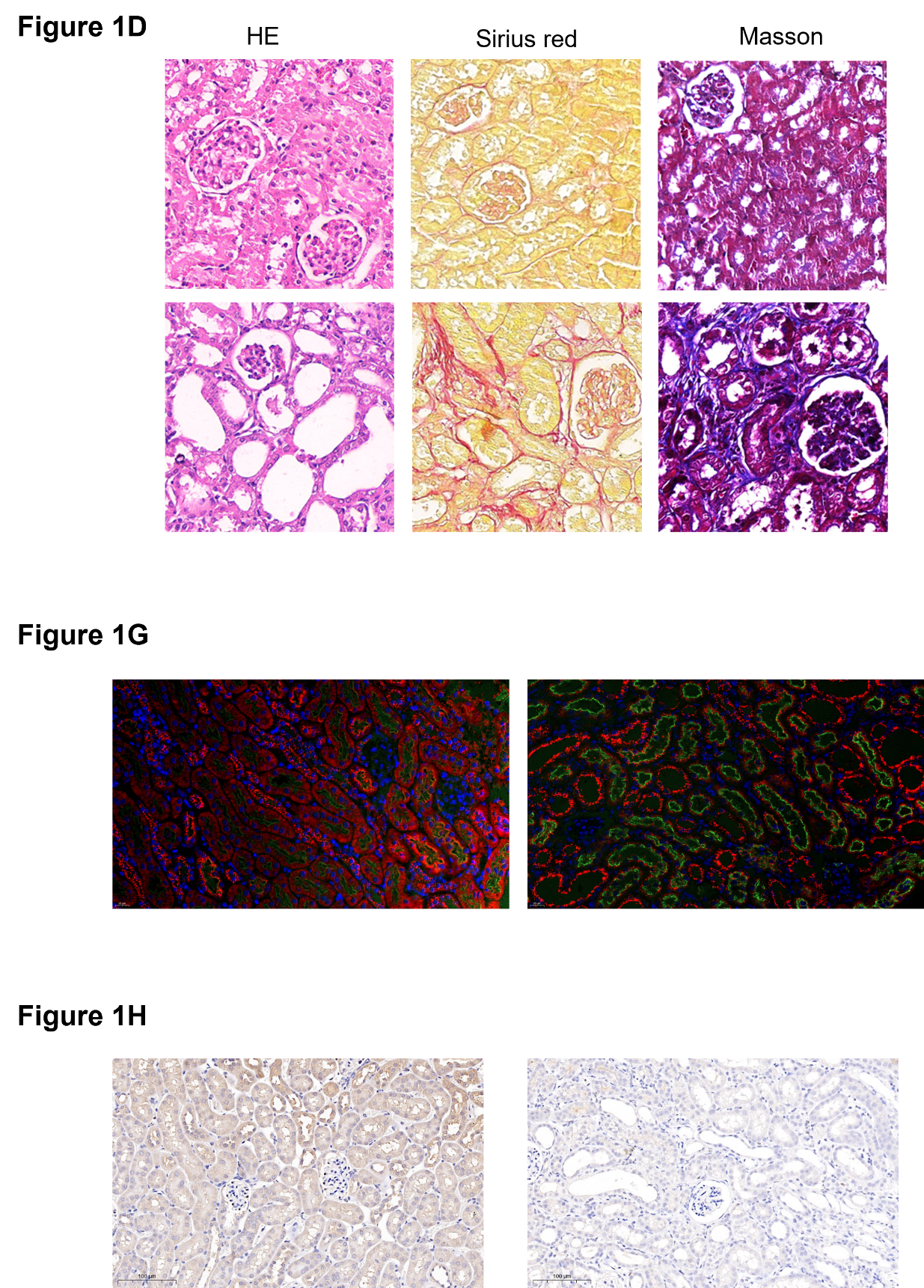 |
| --- |
| 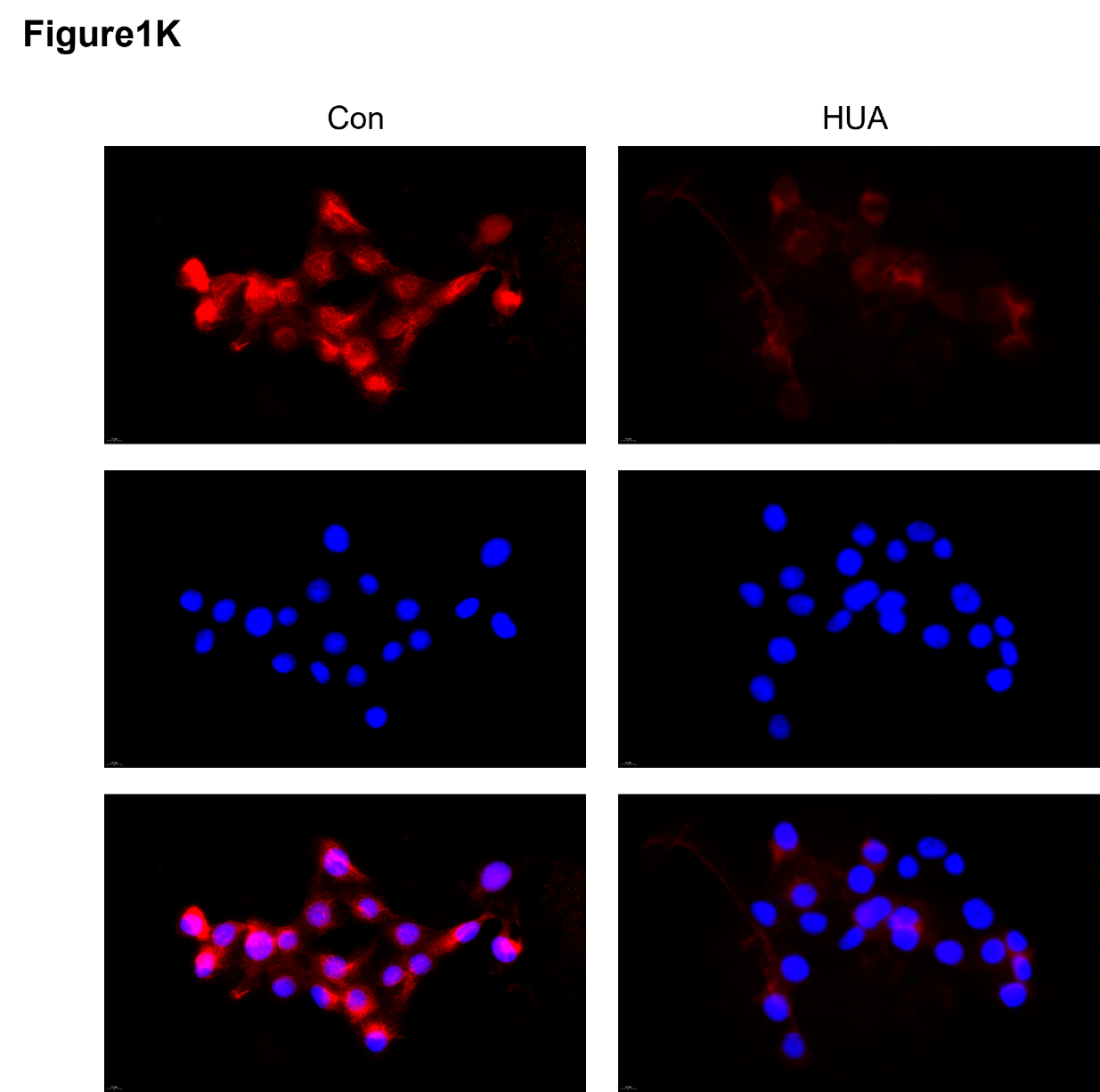 |
| 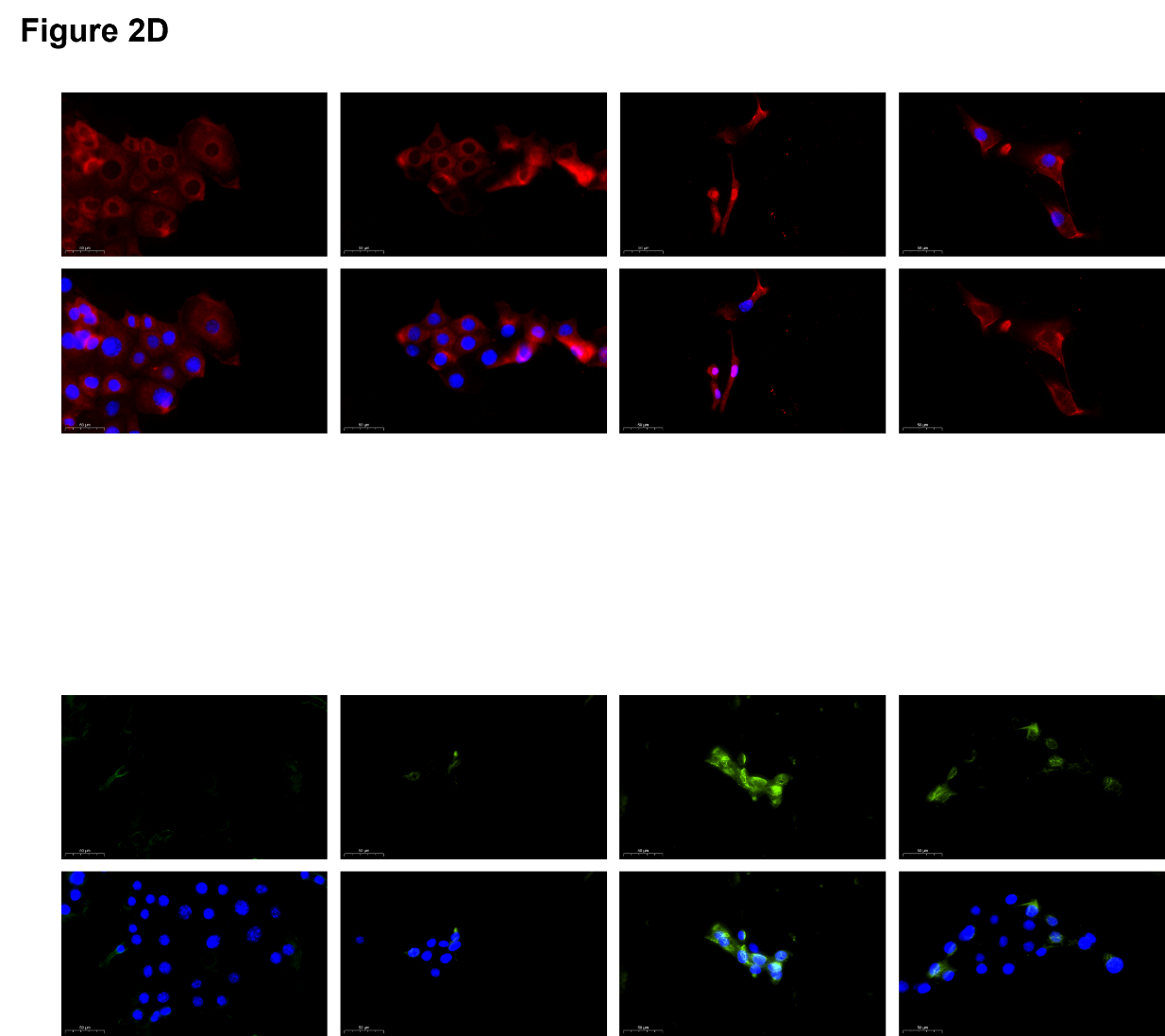 |
| 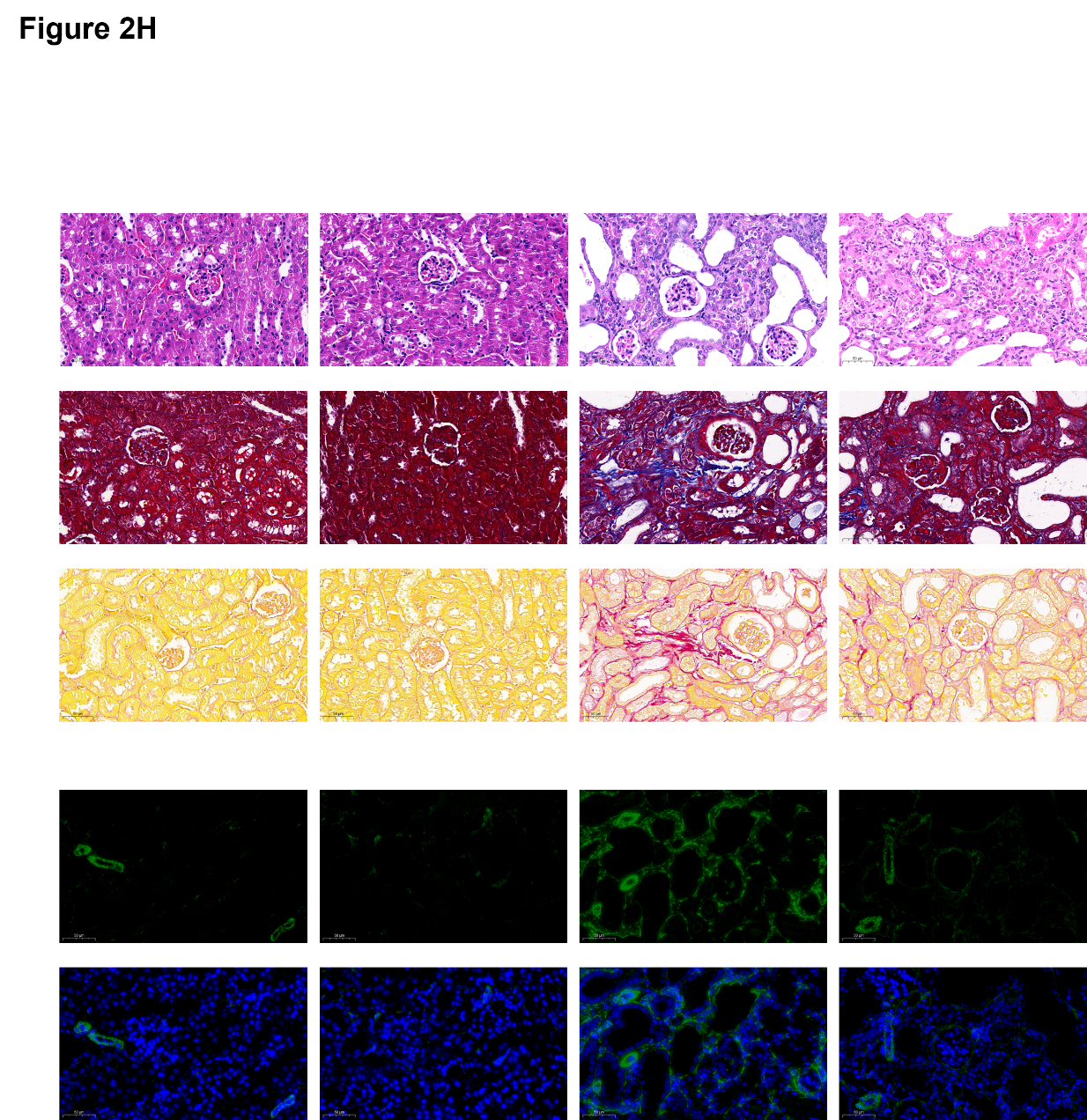 |
| 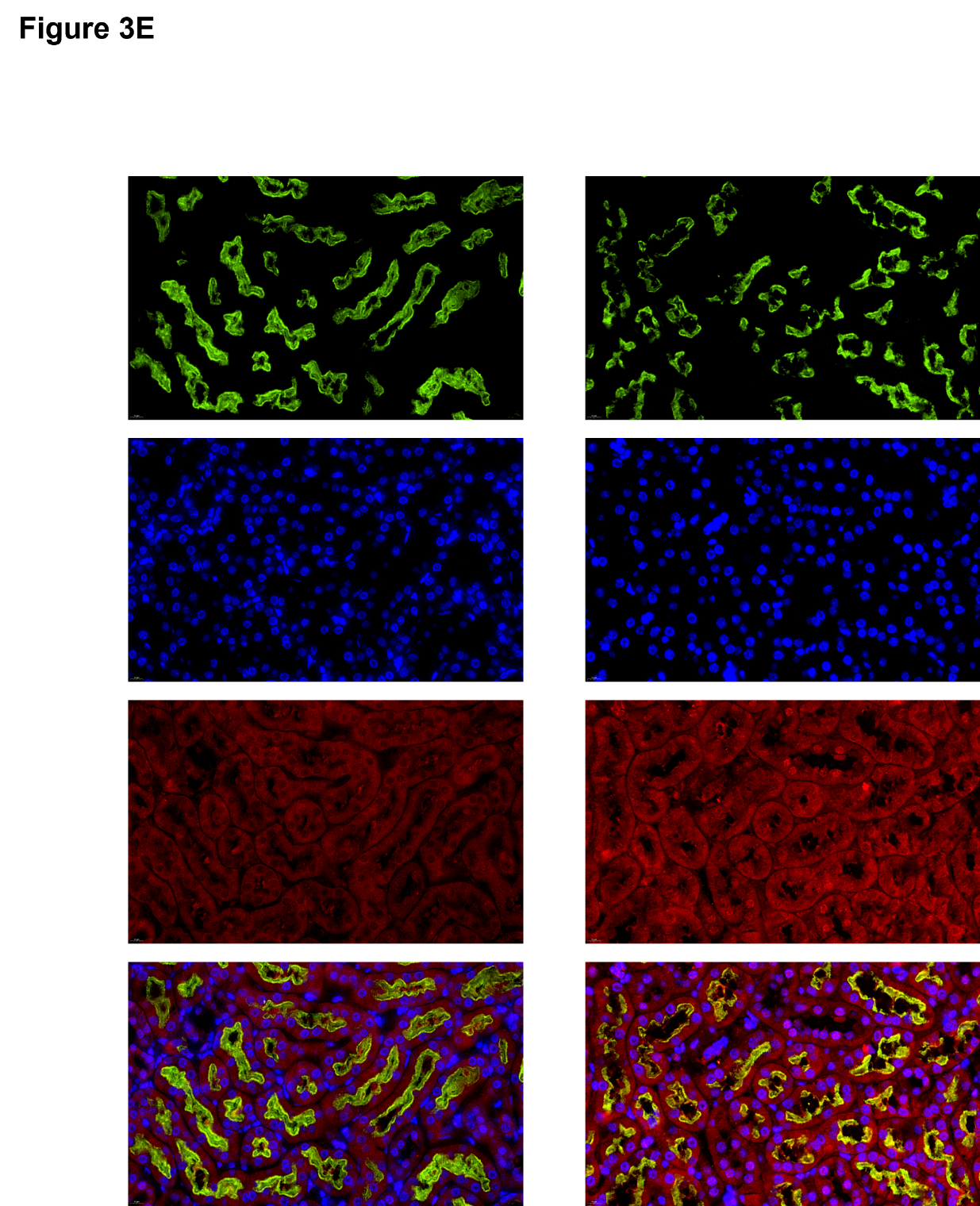 |


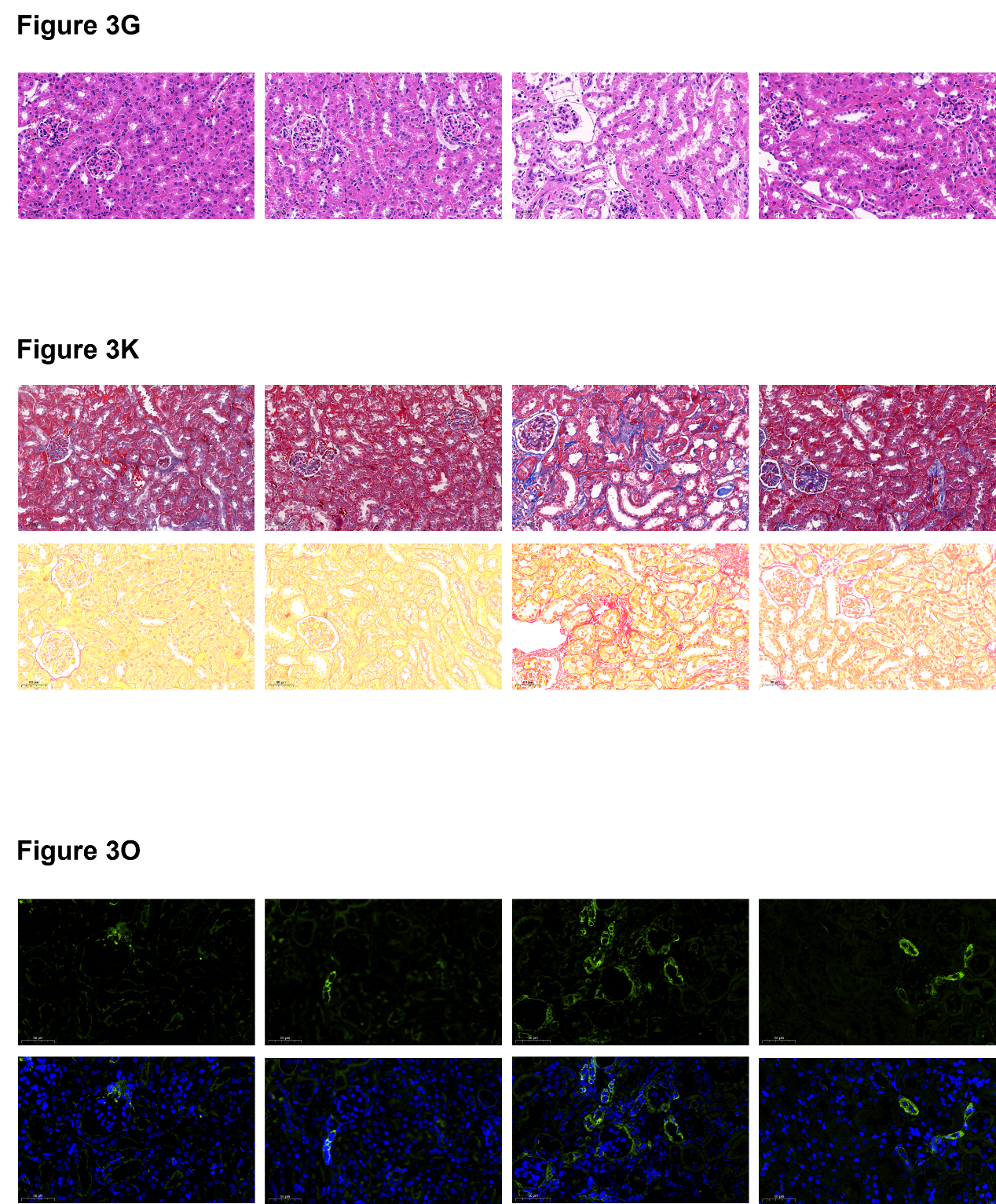

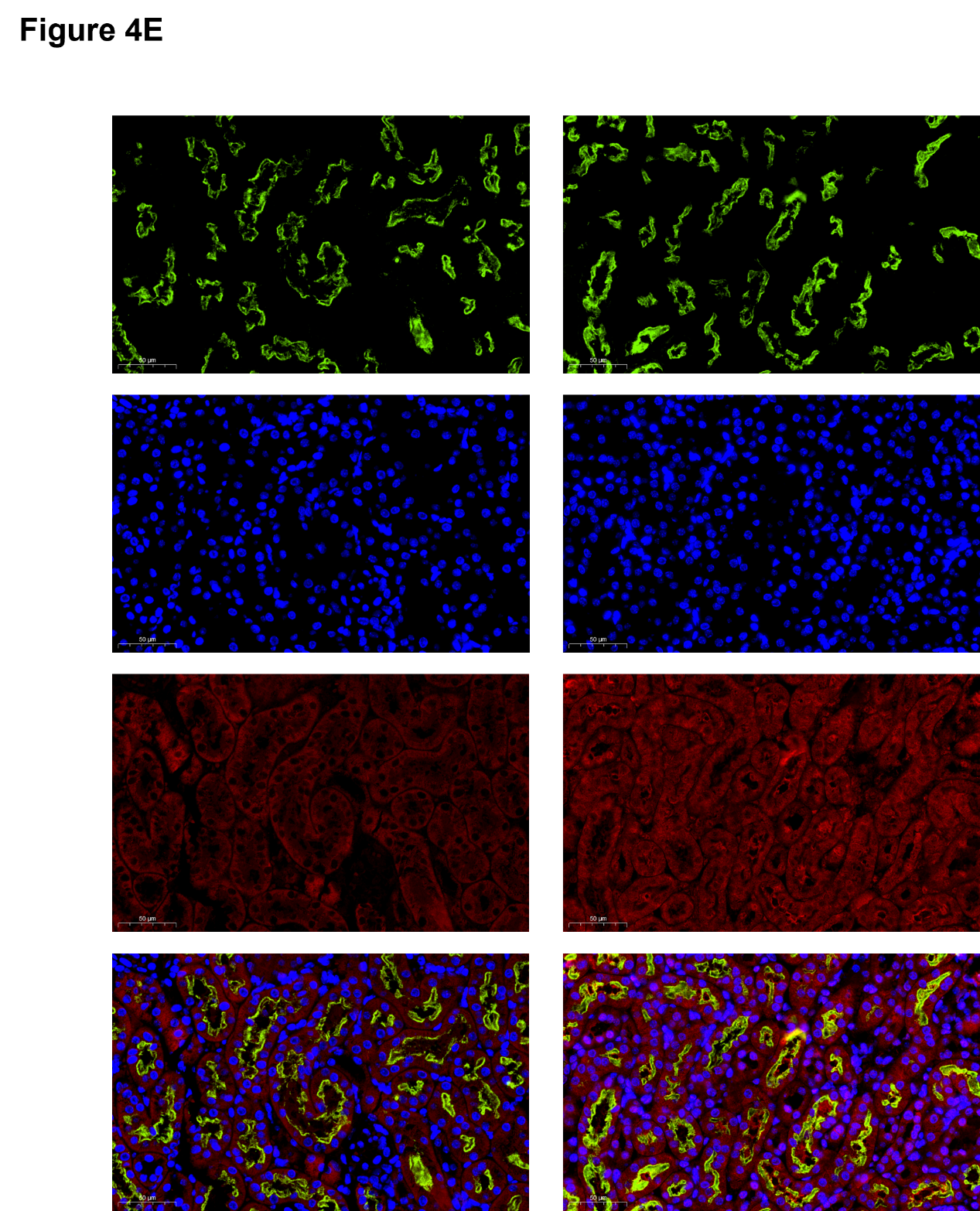

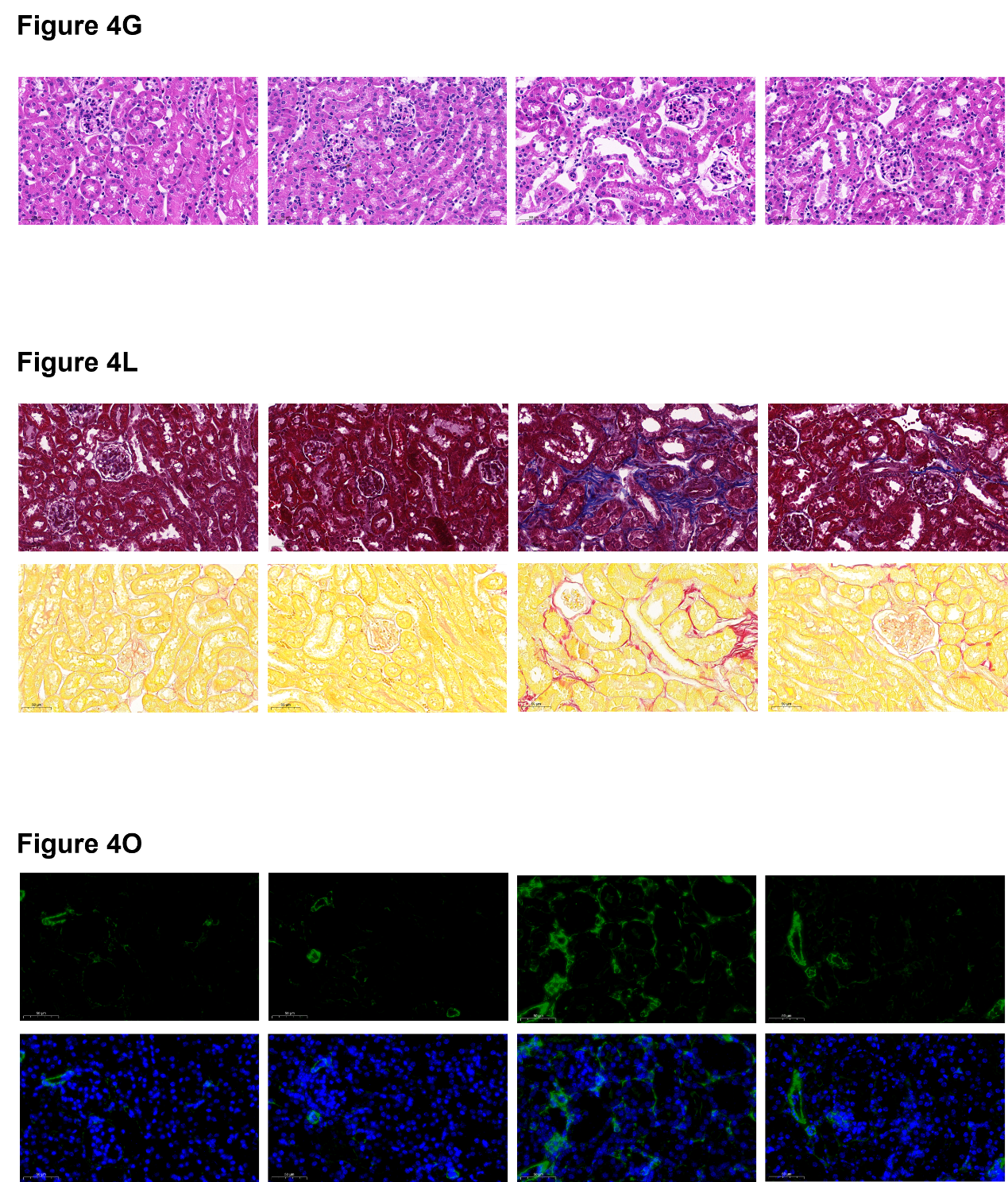

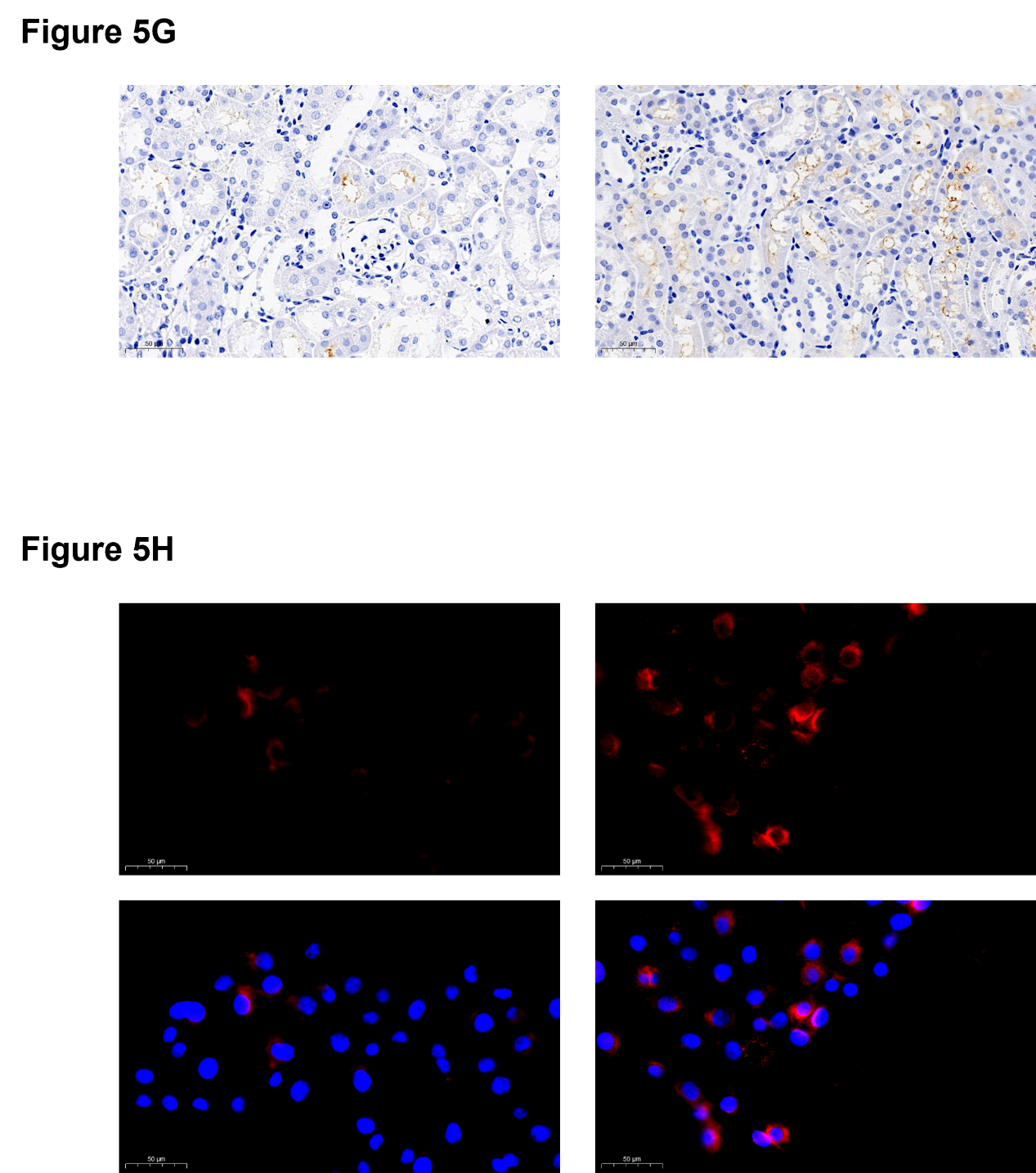

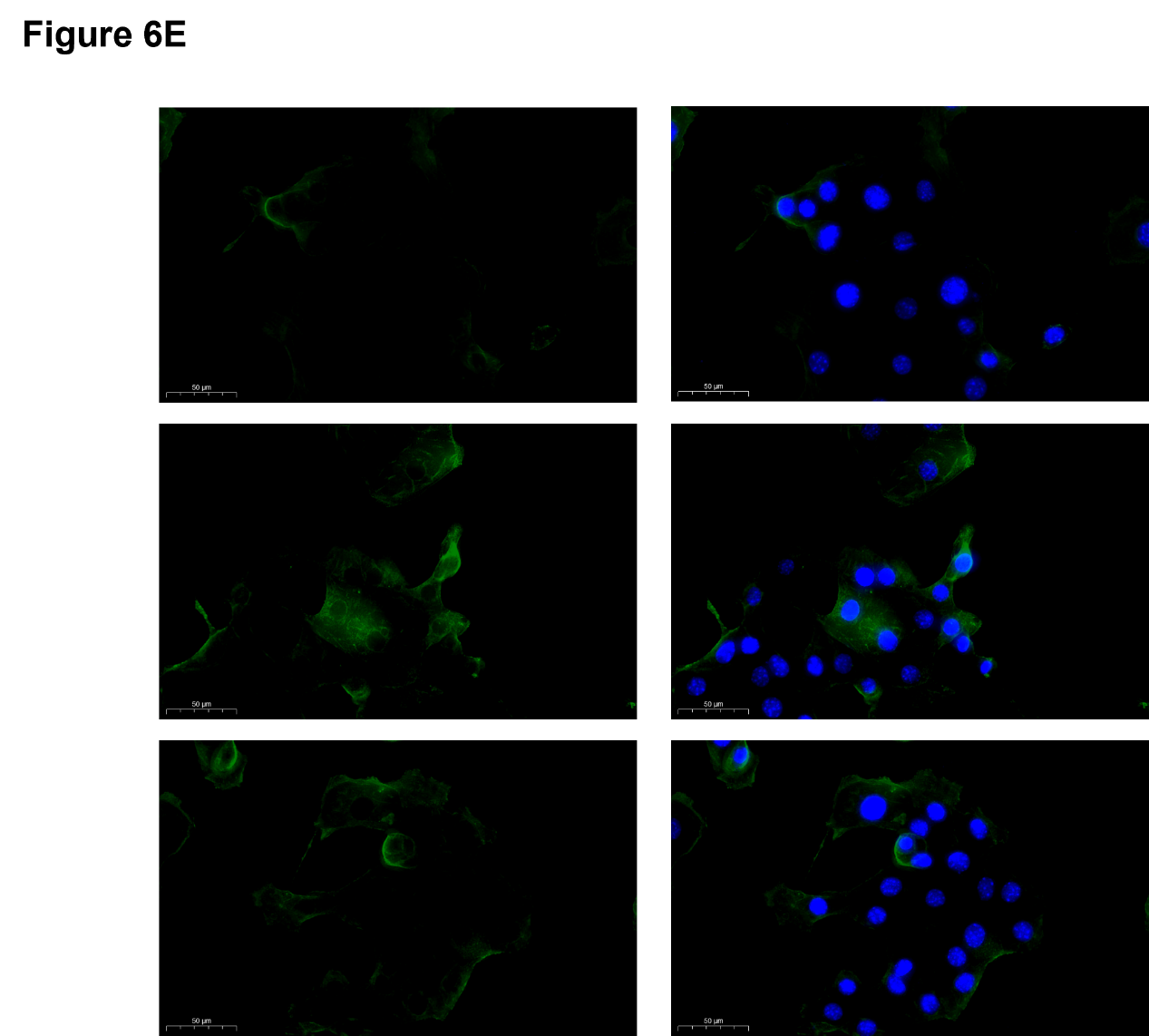

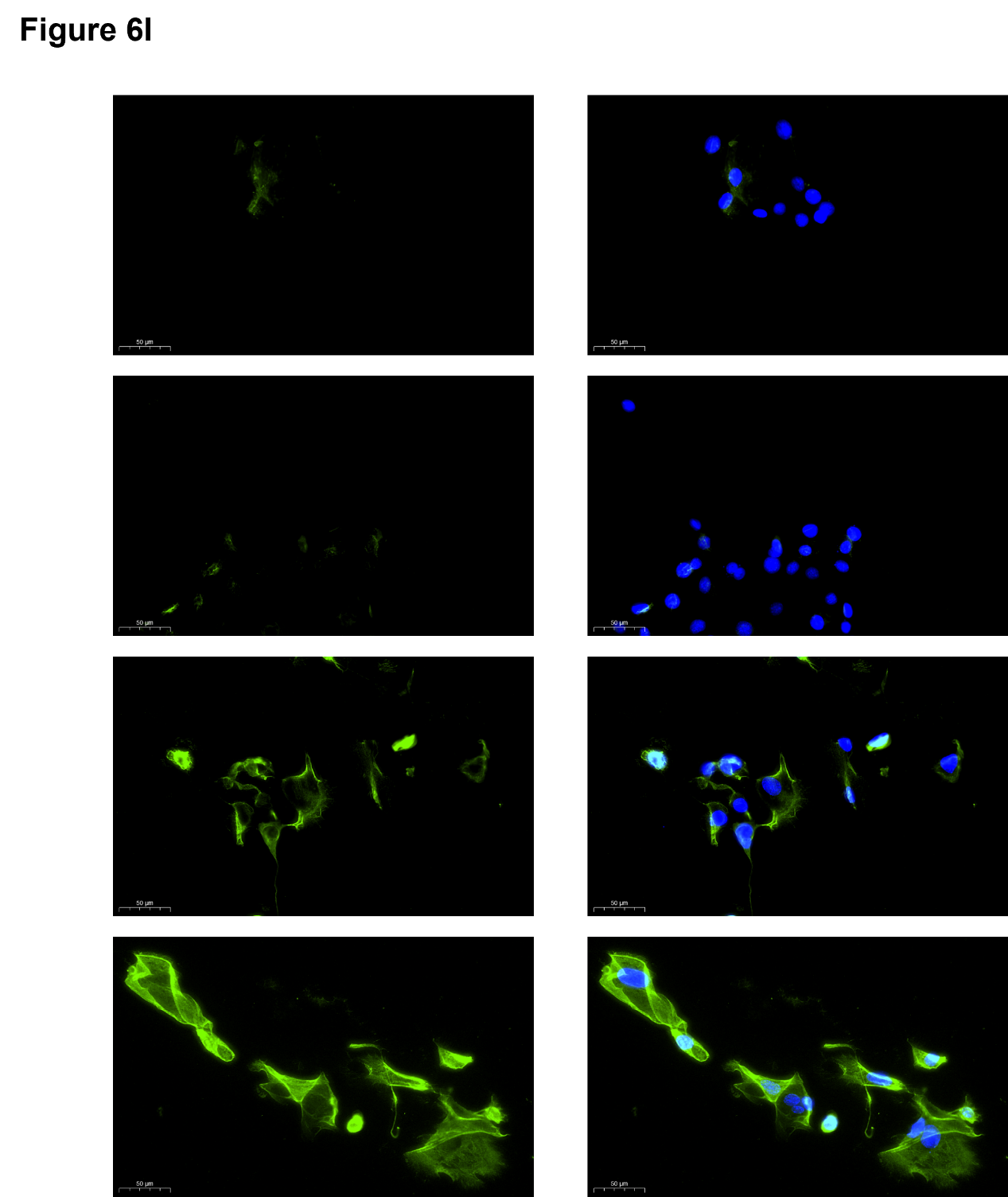

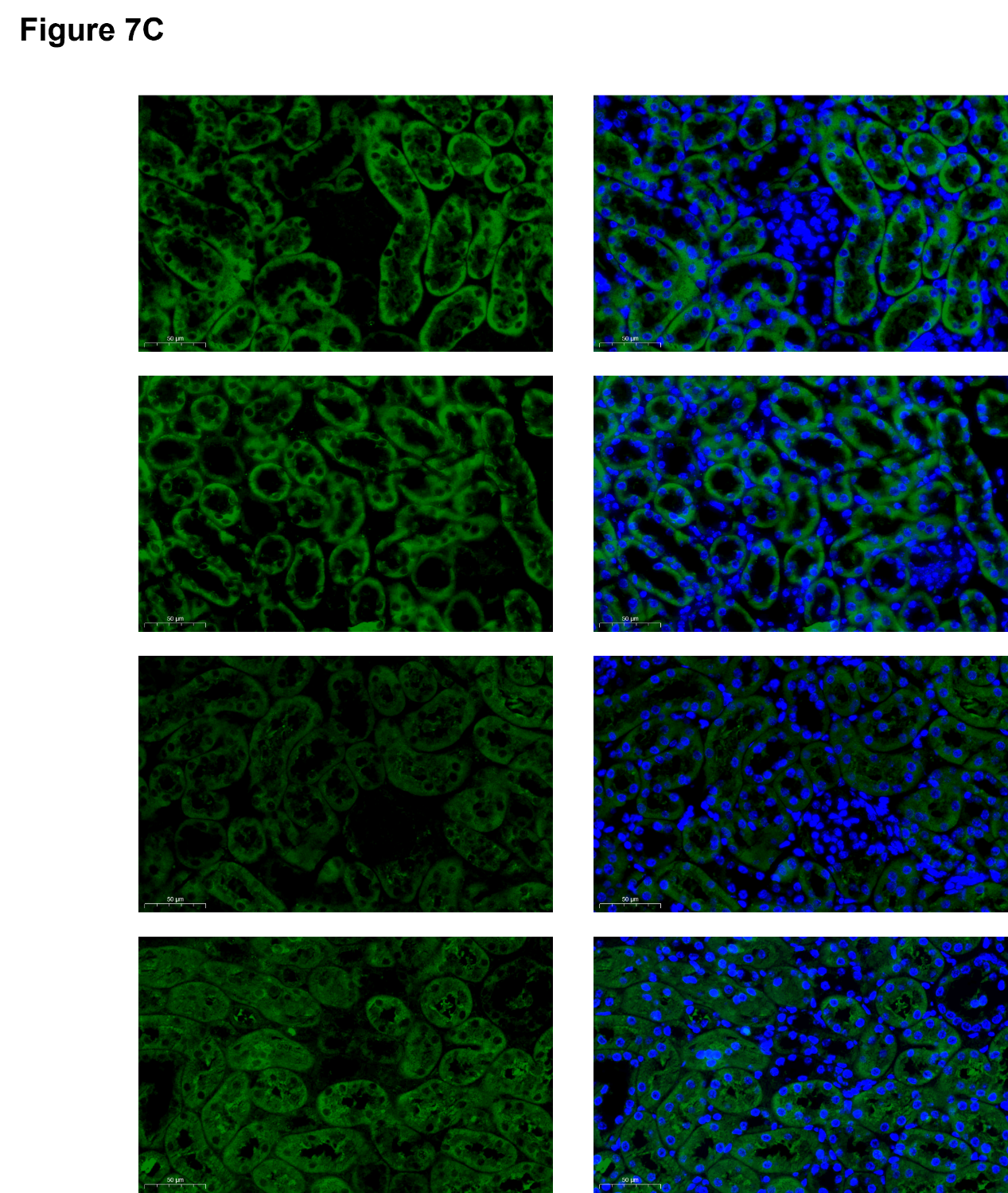

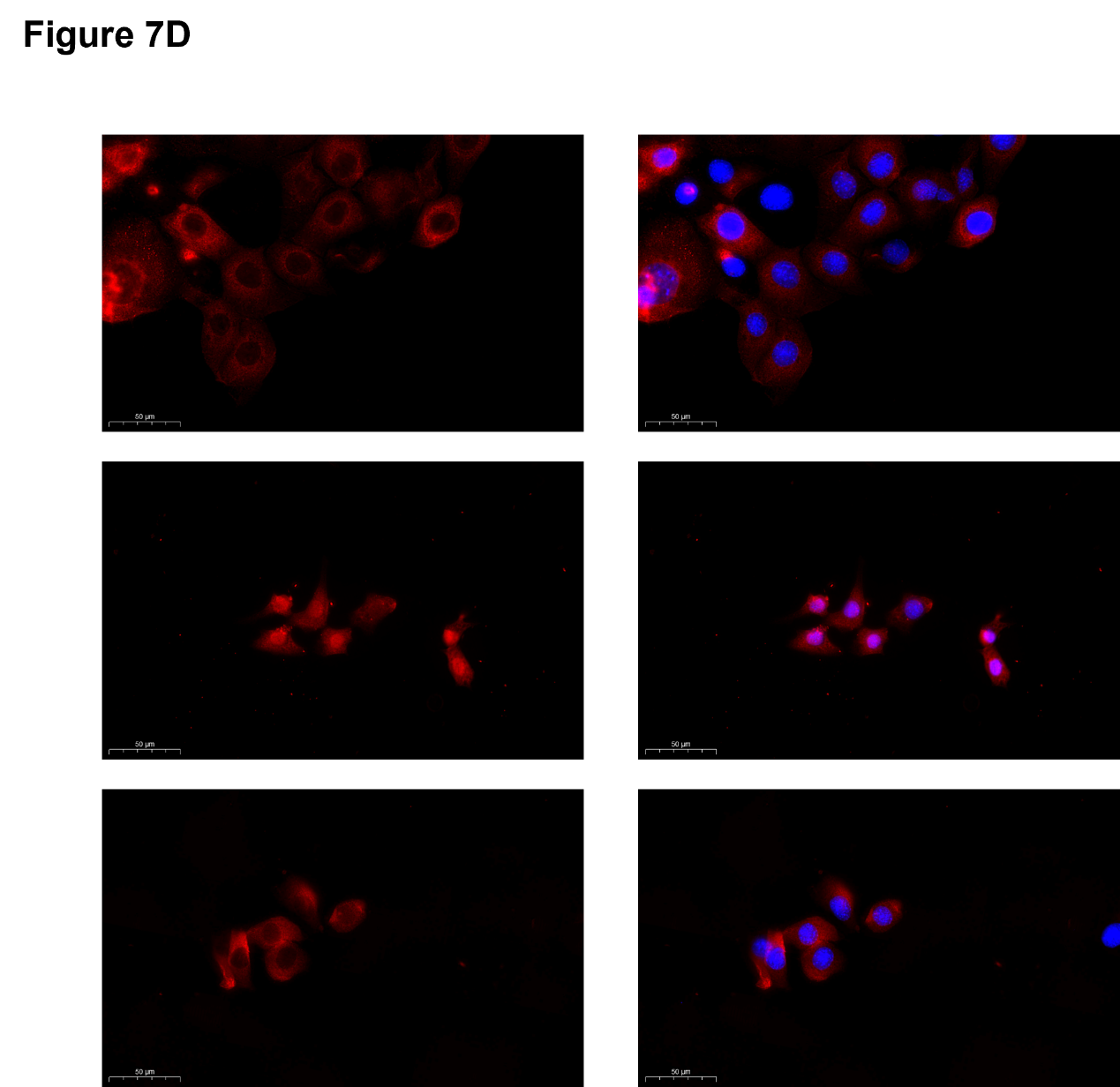

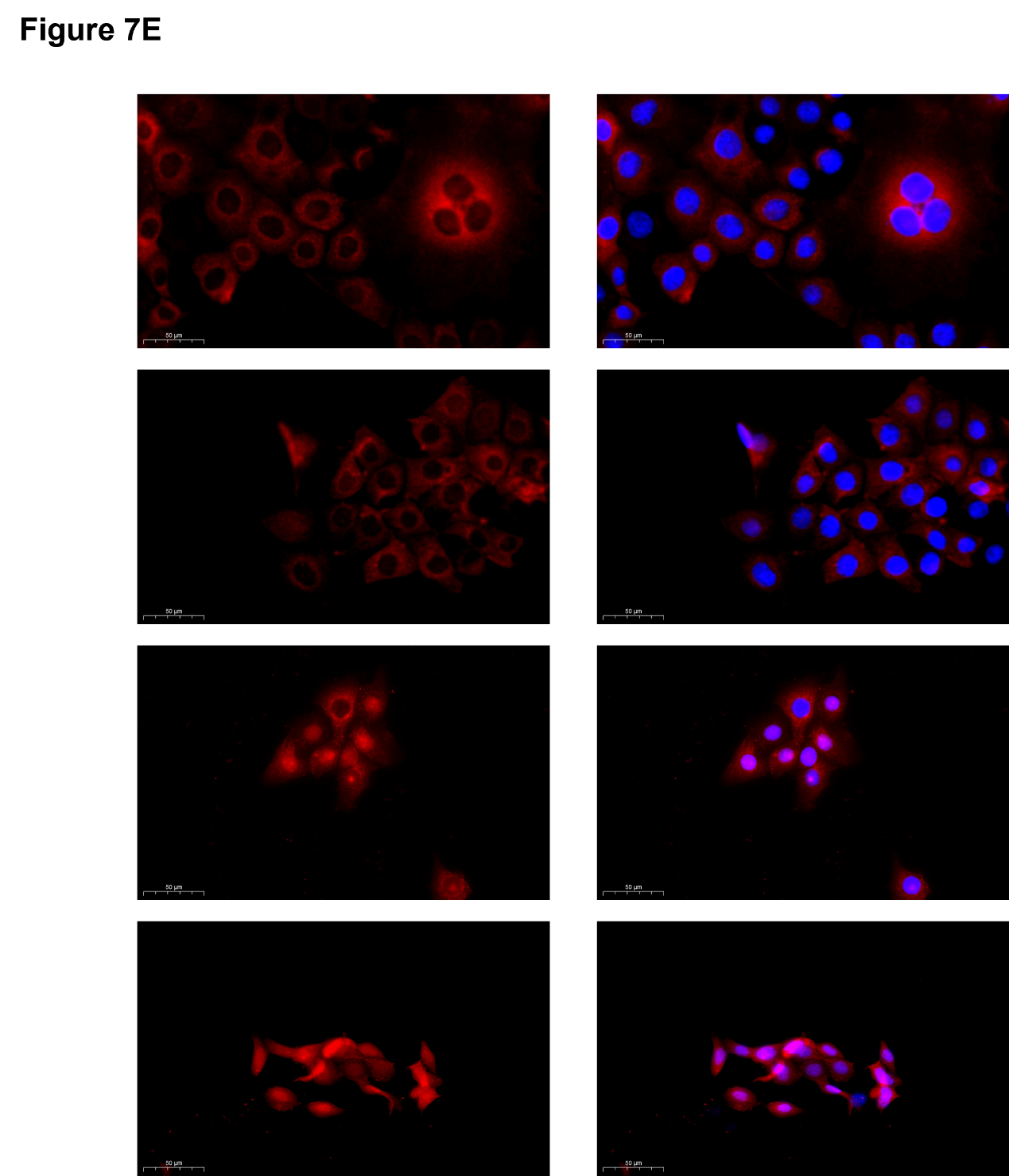

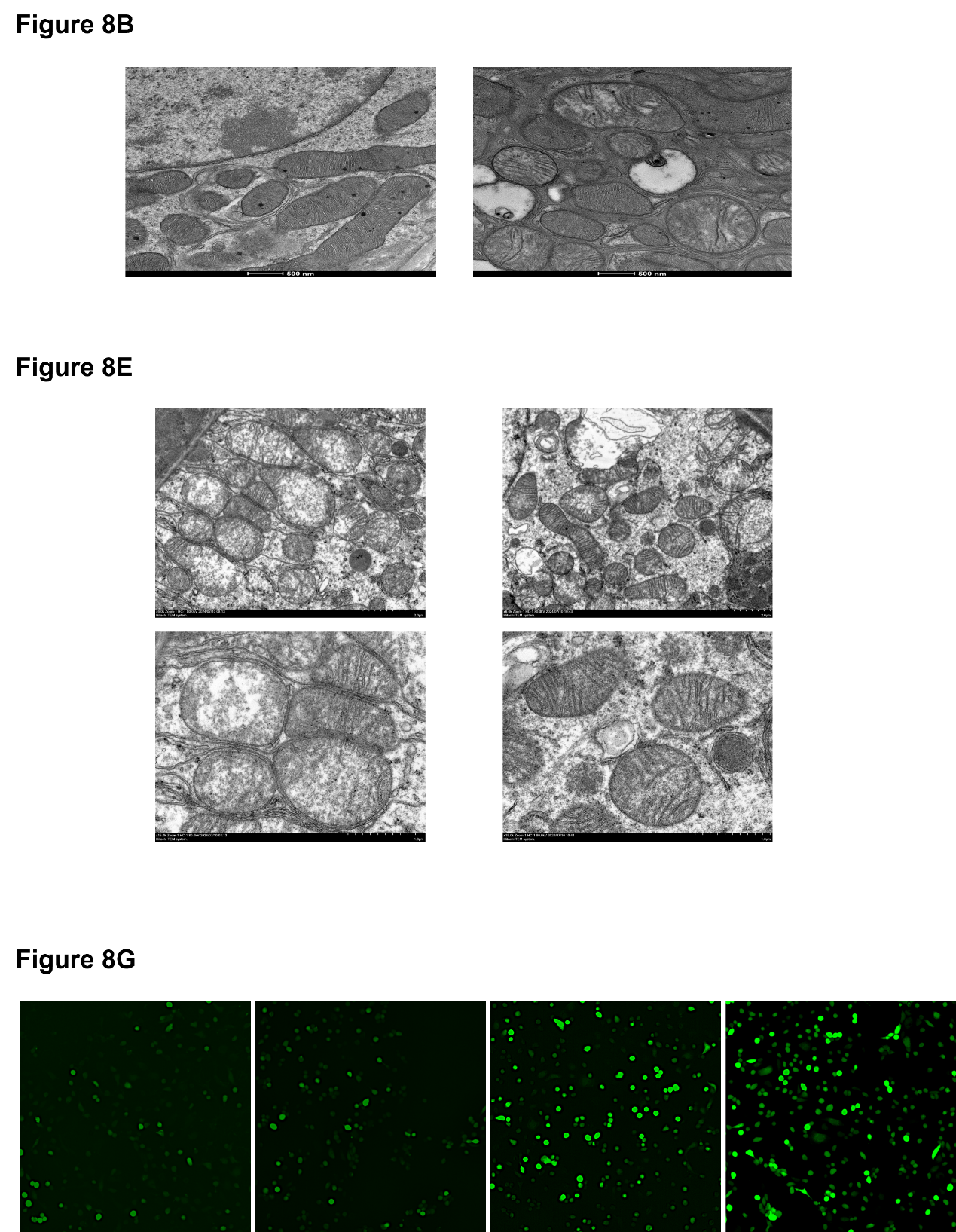

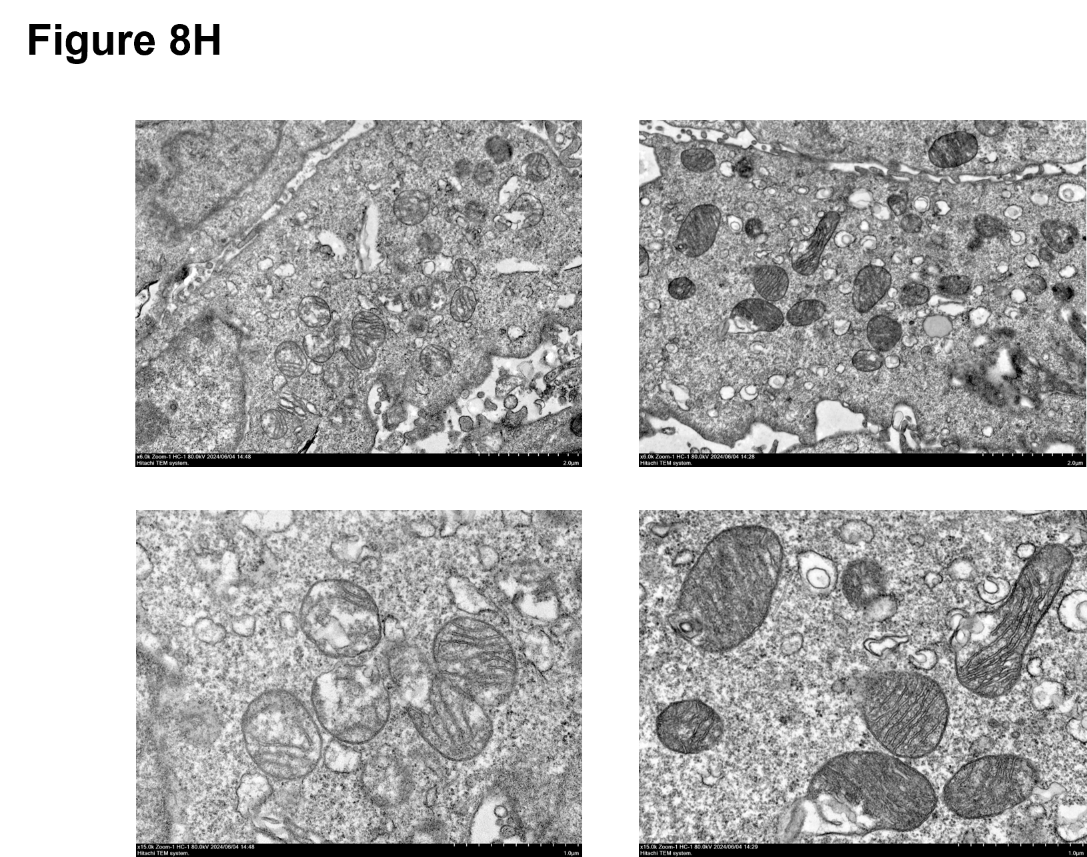

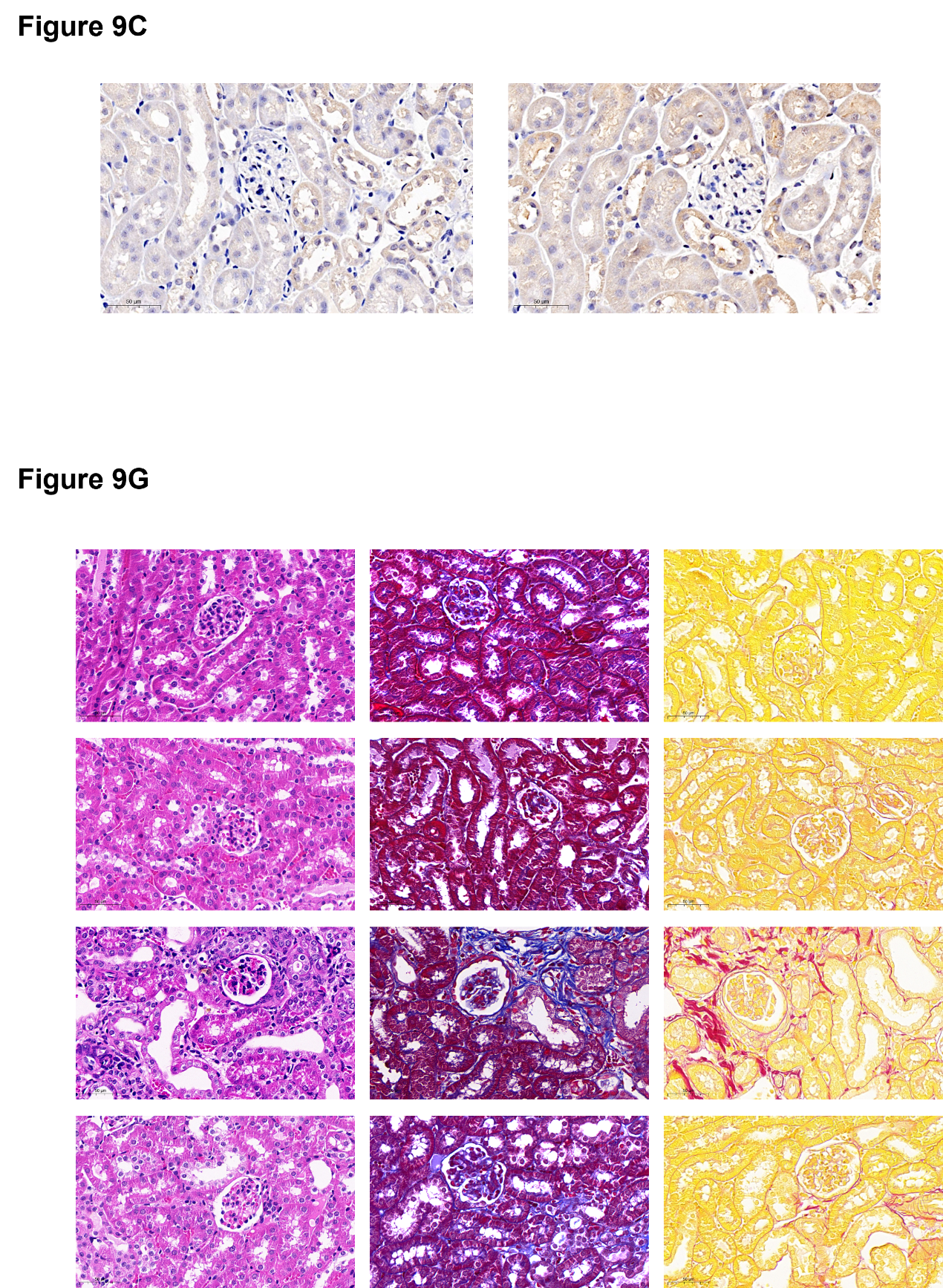

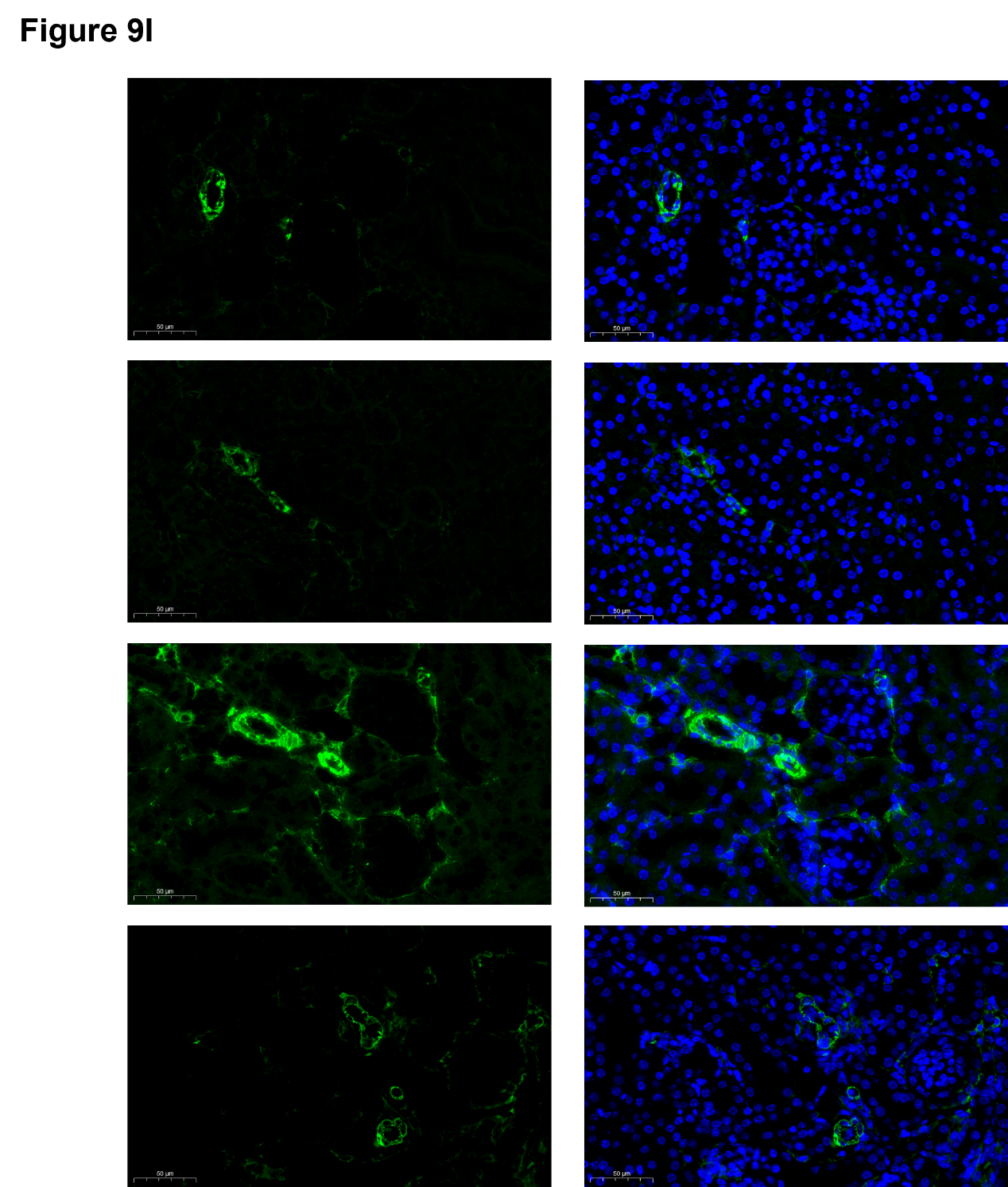

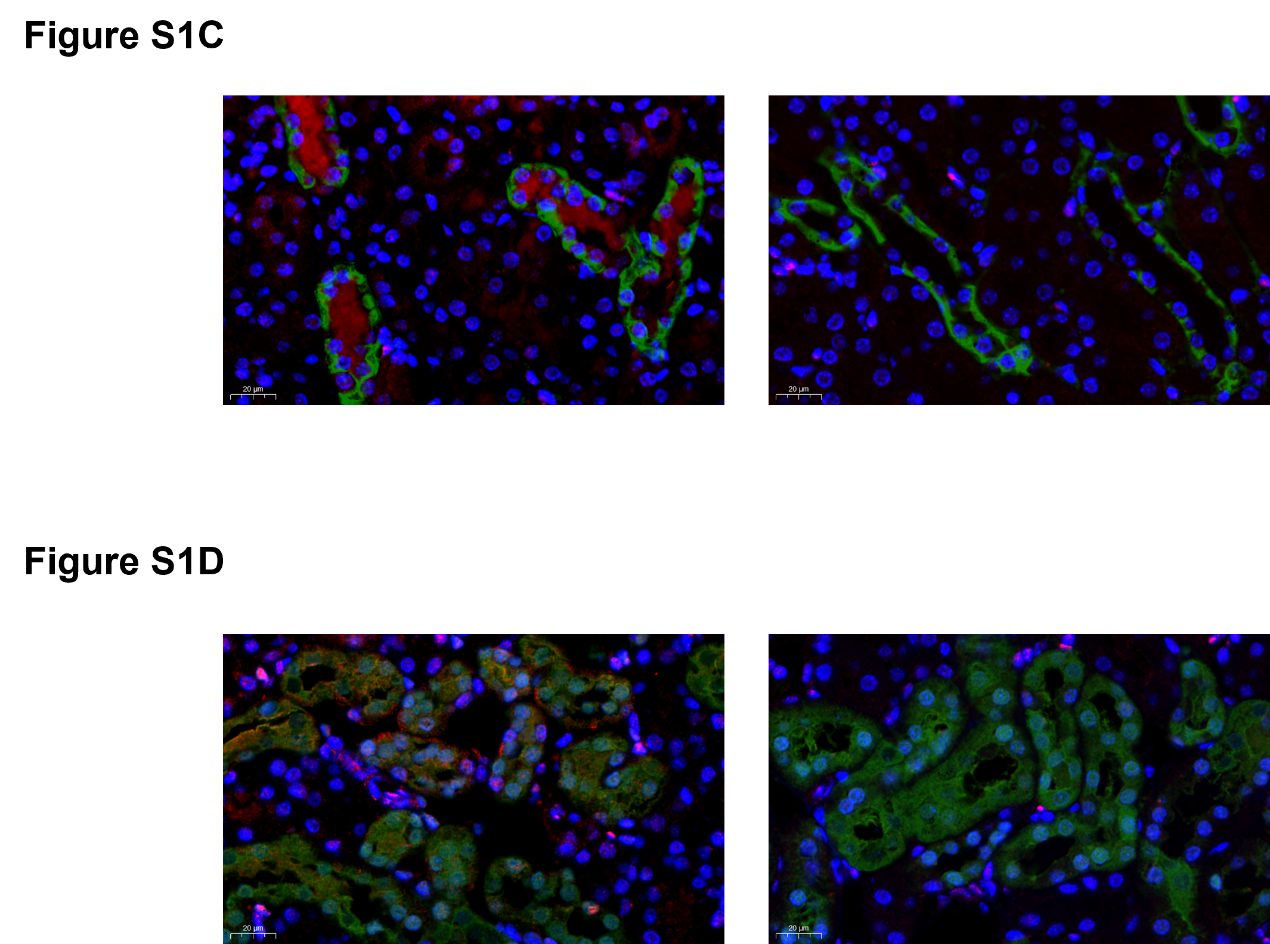

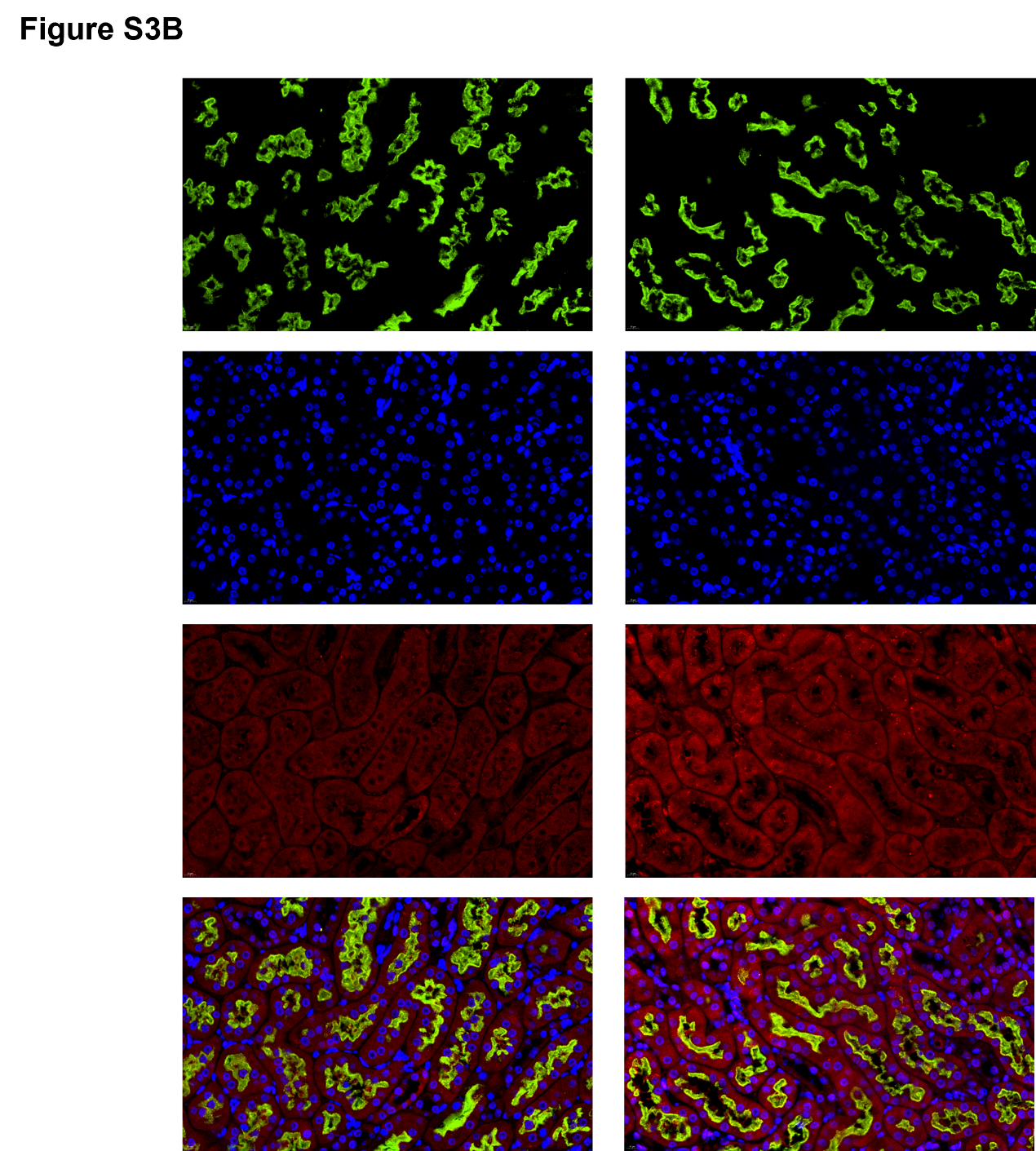

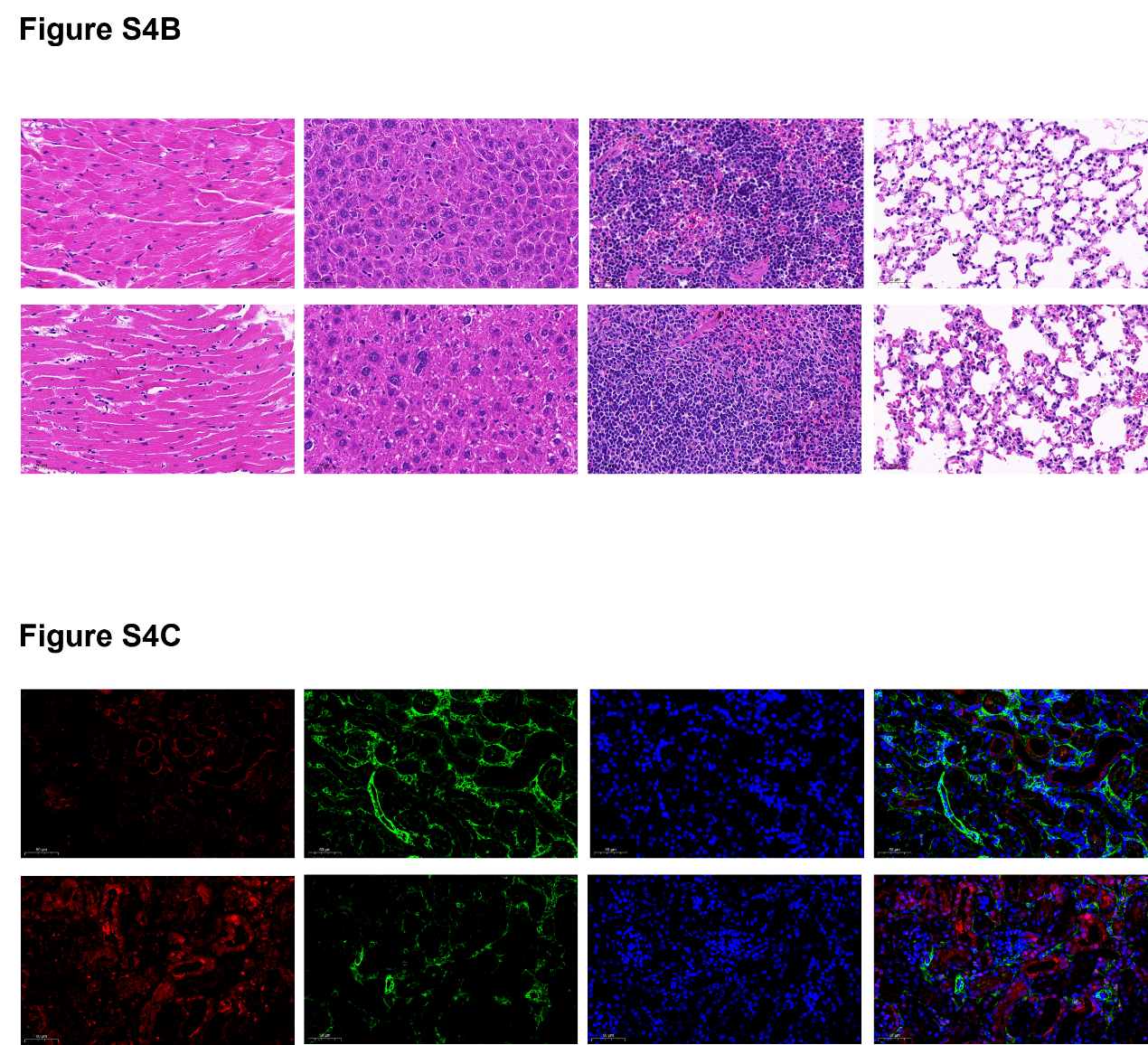

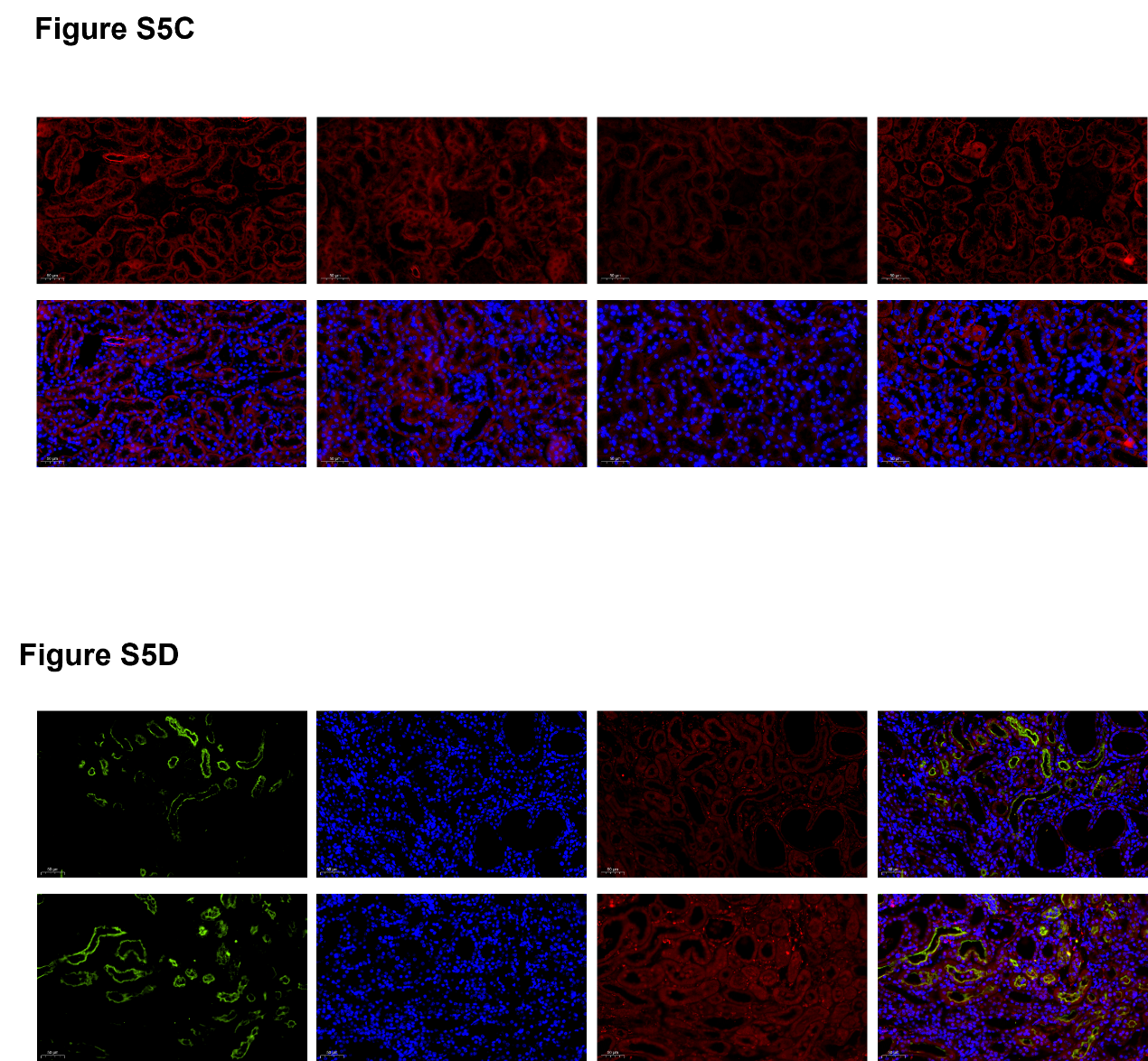


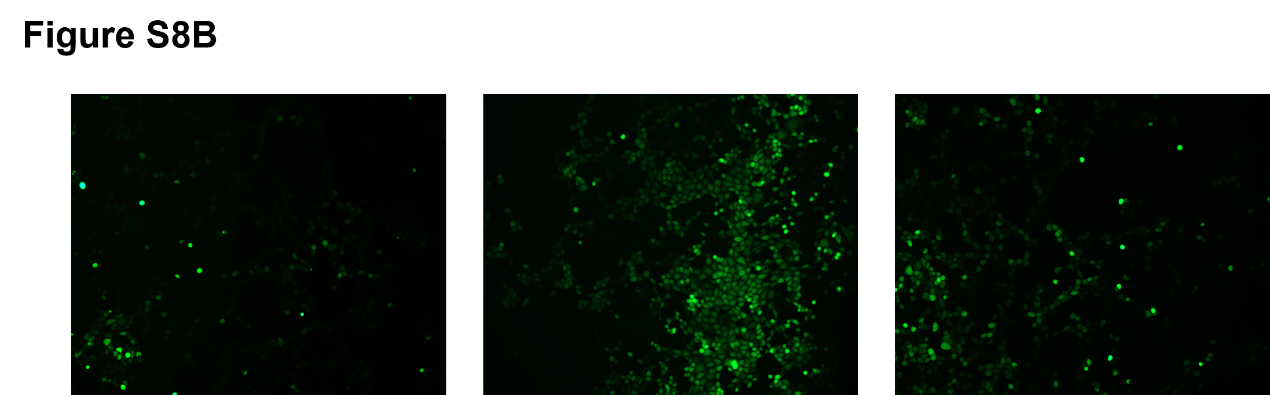

Supplement: Supplementary file 2 — Supporting File 2: advs75092‐sup‐0002‐DataFile.zip. [file ADVS-13-e21459-s002.zip › advs75092-sup-0002-DataFile/Original microscope images.docx]
